# Host metabolites explain microbiome variation between different rice genotypes

**DOI:** 10.1186/s40168-025-02181-z

**Published:** 2025-08-09

**Authors:** Pin Su, Houxiang Kang, Qianze Peng, Weiye Peng, Shu’e Sun, Xiaohua Du, Chi Zhang, Ziling Lei, Lianyang Bai, Qianjun Tang, Yong Liu, Tomislav Cernava, Deyong Zhang

**Affiliations:** 1 State Key Laboratory of Hybrid Rice and Institute of Plant Protection, Hunan Academy of Agricultural Sciences, Changsha, 410125 China; 2 State Key Laboratory for Biology of Plant Diseases and Insect Pests, Institute of Plant Protection, Chinese Academy of Agricultural Sciences, Beijing, 100193 China; 3 Yuelushan Laboratory, Changsha, 410016 China; 4 School of Tropical Agriculture and Forestry, Hainan University, Haikou, 570228 China; 5 Longping Branch, College of Biology, Hunan University, Changsha, 410082 China; 6 College of Plant Protection, Hunan Agricultural University, Changsha, 410128 China; 7 School of Biological Sciences, Faculty of Environmental and Life Sciences, University of Southampton, Southampton, SO17 1 BJ UK

**Keywords:** Plant genotype, Plant microbiome, Genetic control, Plant metabolites, Phenylpropanoids

## Abstract

**Background:**

Plants live in close association with microbial communities that support their health and growth. Previous research has indicated that the composition of these communities can differ between genotypes of the same plant species. Host-related factors causing this variation are mostly unknown. *Microbiome* genes, or *M* genes in short, are host genes that are involved in shaping the microbiome. We hypothesized that specific *M* genes are responsible for microbiome variation between rice genotypes and that it is connected to plant metabolites controlled by these genes.

**Results:**

Our study was aimed at identifying plant metabolites driving genotype-specific microbiome assembly and establishing a link to host genetics. Targeted metabolite quantification was combined with microbiome profiling of the rice phyllosphere microbiome, association analyses on single-nucleotide polymorphism (SNP) level, and genetic modifications to validate microbiome-shaping effects of the discovered *M* genes. Targeted metabolite quantifications revealed that phenylpropanoid concentrations in rice leaves can substantially differ among 110 representative genotypes grown under the same, controlled conditions. Redundancy analyses (RDA) showed that these metabolites can explain 35.6% of the variance in their microbiomes. Further verification experiments resulted in the identification of two *M* genes*. OsC4H2* and *OsPAL06* are both plant genes with microbiome-shaping effects, mainly via their role in ferulic acid biosynthesis. Targeted gene mutation experiments confirm that distinct phyllosphere-associated bacterial groups are highly responsive to the discovered *M* genes.

**Conclusion:**

This study provides detailed insights into the links between host genetics and microbiome variation in plants. Knowledge about host genes that are in control of the microbiome paves the way for microbiome engineering and targeted plant breeding approaches.

Video Abstract

**Supplementary Information:**

The online version contains supplementary material available at 10.1186/s40168-025-02181-z.

## Introduction

Plants are connected to an intricate network of microbes from which they benefit throughout their life cycle [1]. Highly diverse microbial communities inhabiting the plant phyllosphere, rhizosphere, and endosphere are collectively known as the plant microbiome [2]. Plants provide photosynthesized products to their microbial allies [3] and, in return, profit from various microbiome services supporting their growth and health [4–7]. Although much is known about the importance of the microbiome with respect to plant disease resistance [8], nutrient uptake [9], and stress tolerance [10], new discoveries keep challenging our perception of plants as isolated entities. A recent study showed that the rhizosphere microbiome of wheat plants can play a critical role in protecting against viral diseases [11], expanding the knowledge about the protective effects of the microbiome. It even extends to protective effects against leaf-feeding insect pests [12].

Evidence is accumulating that plants actively assemble their microbiome through recruitment or enrichment of specific microbial taxa [13]. This process is crucial to direct microbiome functions towards beneficial effects on plants. For example, the enrichment of the bacterial order Pseudomonadales in rice plants can suppress infection by *Xanthomonas oryzae* [14], while the enrichment of the fungal species *Aspergillus cvjetkovicii* protects against *Rhizoctonia solani* infections [15]. However, our knowledge of how plants assemble their microbiome is patchy and incomplete. It is known that the plant immune system can inhibit the proliferation of pathogenic microbiome members in order to prevent dysbiosis [16]. In addition, increasing evidence indicates that plants possess various molecular mechanisms that allow them to recruit certain microbial taxa, especially those that are beneficial [17, 18]. Plants can use specific metabolites to attract or deter certain microorganisms under different environmental conditions [6, 19, 20], suggesting a vital role of such processes in microbiome assembly. Previous studies have shown that the plant microbiome differs not only from species to species but also from genotype to genotype within the same species [14, 21]. Genotype specificity of plant microbiome assembly has been described for various plants, including *Boechera stricta* [21], sorghum [22], barley [23], tomato [24], and *Arabidopsis thaliana* [25]. A genome-wide association study (GWAS) linking bacterial community structures on rice leaves identified 2667 non-redundant SNPs located in 235 loci providing insights into the potential extent of host genetic control of the microbiome [14]. In addition to microbiome variation, it is also known that different plant genotypes can be associated with different levels of certain metabolites [26]. Plant specialized metabolites can have selective growth modulation activities toward microbiota members [3]. Various plant metabolites are known to selectively modulate the growth of distinct members [3]. This led us to speculate that variation in phyllosphere microbiomes among different rice genotypes is caused by differences in their individual compositions of microbiome-shaping metabolites. A foregoing study suggested implications of the phenylpropanoid biosynthesis pathway for metabolite-driven microbiome assembly [14]. In the phenylpropanoid biosynthesis pathway, l-phenylalanine (LPA) serves as the initial substrate, which is then converted into different aromatic compounds. The products include lignin, coumarins, lignans, flavonoids, and stilbenes, which are used by plants in numerous biological processes [27] and are potentially relevant for microbiome assembly processes.

In this work, we quantified five phenylpropanoids and one phenylpropanoid derivative in leaf tissues of 110 rice varieties of the Rice Diversity Panel II core collection (C-RDP-II) [28]. We used the same plant varieties for microbiome profiling and showed that both phenylpropanoid concentrations and microbiome assembly were specific to rice genotypes. Computational analyses provided the first evidence that phenylpropanoids were responsible for substantial phyllosphere microbiome variation among different rice genotypes. Sequence comparison of gene haplotypes, comparison of metabolite concentrations, and abundance analysis of specific taxa between haplotypes showed that nucleotide variations of two genes *OsC4H2* and *OsPAL06* from the phenylpropanoid biosynthesis pathway were significantly linked to ferulic acid concentrations as well as the abundances of the prevalent microbiome members Burkholderiales. To verify the implications of host genetics on metabolite-driven microbiome assembly, we implemented knockout mutants and overexpression constructs of the two genes. Our results showed that *OsC4H2* and *OsPAL06* were involved in genotype-specific microbiome shaping and thus are *M*icrobiome genes, or *M* genes in short, as introduced recently [14]. Overall, our study provides detailed explanations for plant genotype specificity of the microbiome.

## Materials and methods

### Chemical reagents and analytical instruments

The chemical reagents and analytical instruments used in this study are listed as follows: ethanol (EtOH), sodium hypochlorite (NaOCl), Triton X-100, 4-hydroxycinnamic acid (HCA), l-phenylalanine (LPA), ferulic acid (FLA), caffeic acid (CFA), scopoletin (SPLT), sinapic acid (SPA), graphitized carbon black (GCB), ammonium formate, and acetonitrile (ACN) were purchased from Macklin (Shanghai, China); formic acid was purchased from ROE Scientific (Newark, USA); agar powder, agarose gel, PBS buffer, and Murashige and Skoog (MS) medium were purchased from Solarbio (Beijing, China); MagPure Soil DNA LQ Kit was purchased from Magen (Shanghai, China); Agencourt AMPure XP beads were purchased from Beckman Coulter (Pasadena, USA); and Qubit dsDNA assay kit was purchased from Yeasen (Shanghai, China).

The analytical equipment used included an ultra-high performance liquid chromatography instrument (ACQUITY UPLC H-CLASS, Waters, USA) coupled to a triple quadrupole mass spectrometer (ACQUITY TQD, Waters, USA) equipped with an electrospray ionization (ESI) source (LC–MS), and a NanoDrop ND-1000 spectrophotometer (Thermo Fisher Scientific, USA).

### Plant material, seed germination, and growth conditions

Seeds of the 110 rice varieties used for profiling and the variety ZH11 used for genetic manipulation in this study were obtained from Hunan Hybrid Rice Research Center (HHRRC, Changsha, China; rice variety information is provided in Dataset 1). ZH11 was implemented as the wild-type (WT) and to generate *c4h2* (*OsC4H2*-knockout), *OsC4H2*-OE (*OsC4H2*-overexpression), *pal06* (*OsPAL06*-knockout), and *OsPAL06*-OE (*OsPAL06*-overexpression) plants. Plants were cultured under relative humidity set at 80%, temperature at 28 °C, and a 13-h light cycle in a greenhouse.

Rice plants were grown in a substrate consisting of field soil mixed with greenhouse potting soil (PINDSTRUP substrate, Denmark; autoclaved twice) in an air-circulating greenhouse. For substrate preparation, soil was collected from a rice field in Taojiang County (28°38′09″ N, 112°0′57″ E), Hunan Province, China. The top 10–20 cm of field soil was collected and sieved (3-mm sieve) to remove rocks and other debris and dried at room temperature. Then, the soil was mixed with sterile potting soil (1:1 w/w), followed by supplementation with sterile half-strength Murashige and Skoog (MS) medium solution (pH 5.8) (1:1 w/v). Rice seeds were surface-sterilized using 75% ethanol (EtOH) for 2 min and 7% sodium hypochlorite (NaOCl) supplemented with 0.2% Triton X-100 3 times for 8 min, following washing with sterile water 6 times. Sterile seeds were then immersed in sterile water for 1 day at 4 °C in the dark. Subsequently, all rice seeds were sown into the pots prepared with the aforementioned soil. Each plot was watered with 50 mL of sterile water twice a week and sterile half-strength MS solution once a week after germination. The top leaves of 5-week-old plants were removed using sterile scissors (surface-sterilized using 75% EtOH and washed with sterile water 6 times for each plant) for the collection of phyllosphere bacteria and the extraction of leaf metabolites.

### Generation of CRISPR-edited and gene overexpression rice mutants

The CRISPR–Cas9 system was used to generate *OsC4H2* (Os05g25640) and *OsPAL06* (Os02g41680) mutants according to a high-efficiency monocot genome-editing method [29]. Briefly, specific primers containing the *OsC4H2* and *OsPAL06* target site sequences (CCTCGTGGAGAAGGTCCTCCTGG) and (GCTGAACTGGGGGAAGGCCACGG) were ligated into pEGCas9Pubi-H cassettes, respectively. To construct guide RNA genes, the rice small nuclear RNA U6 promoter was amplified from the pYLsgRNA-OsU6a vector [29] using primer pairs U6-1/U6-2. The *OsC4H2*-gRNA and *OsPAL06*-gRNA scaffolds were amplified from the pYLsgRNA-OsU6a vector [29] using primer pair gRNA-c4h2/gRNA-2 and gRNA-pal06/gRNA-2, respectively. The PCR product of the U6 promoter was fused with the *OsC4H2*-gRNA or *OsPAL06*-gRNA scaffold by overlapping PCR using primer pair sgRNA-1/sgRNA-2. The U6 promoter-gRNA fragments were cloned into the pEGCas9Pubi-H vector to form the pEGCas9Pubi-H-Os05g25640 and pEGCas9Pubi-H-Os02g41680 constructs via BsaI (Takara, Beijing, China) restriction digestion followed by ligation using T4 DNA Ligase (Takara, Beijing, China), respectively. After transformation into *Escherichia coli* DH5-alpha, the resulting constructs were purified with the TIANprep Plasmid Midi kit (TIANGEN, Beijing, China) for PCR amplification using primer pairs EGCas-1/EGCas-2 and sequenced via Sanger sequencing (Sangon Biotech, Shanghai, China) (Fig. S1). The validated constructs were stored at − 20 °C for subsequent use in rice protoplast transformation.

The *OsPAL06*-overexpression and *OsC4H2*-overexpression constructs were generated according to a transgenic rice construction method [30]. Briefly, for the *OsPAL06* and *OsC4H2* overexpression vector constructs, total RNA of rice variety ZH11 leaves was extracted using TRIzol, and complementary DNA (cDNA) was synthesized from the total RNA using the cDNA Synthesis SuperMix kit (TransGen Biotech, Beijing, China). The *OsC4H2* and *OsPAL06* coding sequences were amplified by PCR using primer pairs c4h2-1/c4h2-2 and pal06-1/pal06-2, respectively. The amplified *OsC4H2* and *OsPAL06* fragments were cloned into the pEGOEPubi-H vector, which contained the maize ubiquitin promoter and a hygromycin resistance gene, to form the pEGOEPubi-H-Os05g25640 and pEGOEPubi-H-Os02g41680 overexpression construct via BsaI (Takara, Beijing, China) restriction digestion followed by ligation using T4 DNA Ligase (Takara, Beijing, China), respectively. After transformation into *Escherichia coli* DH5-alpha, the resulting constructs were purified with TIANprep Plasmid Midi kit (Tiangen, Beijing, China) for PCR amplification using primer pairs EGOEPubi-1/EGOEPubi-2 and sequenced via Sanger sequencing (Sangon Biotech, Shanghai, China) (Fig. S2). The validated constructs were stored at − 20 °C for subsequent use in rice protoplast transformation.

The resulting vectors were introduced into *Agrobacterium tumefaciens* (strain EHA105) and then into rice by *Agrobacterium*-mediated transformation [31]. The transformation was performed using a specific rice transformation method [31]. Briefly, positive transgenic bacteria were selected by using kanamycin for selection. Hygromycin was used to select putative transgenic plants. All the selected putatively transgenic seedlings were cultivated in the greenhouse or field for further identification.

To identify transgenic plants, genomic DNA was extracted from approximately 30 mg of leaf tissue using FastPure Plant DNA Isolation Mini Kit (Vazyme Biotech, Nanjing, China) according to the manufacturer’s instructions. The isolated and purified genomic DNA was used for PCR amplification of CRISPR/Cas9 *OsC4H2* and *OsPAL06* target sites or hygromycin resistance gene using primer pairs c4h2ko-1/c4h2ko-2 and pal06ko-1/pal06ko-2 or H-1/H-2, respectively. Amplified fragments were detected using agarose gel electrophoresis, purified using EasyPure® Quick Gel Extraction Kit (TransGen Biotech, Beijing, China), and sequenced with Sanger sequencing (Sangon Biotech, Shanghai, China) to identify *c4h2*, *pal06*, *OsC4H2-OE*, and *OsPAL06-OE* transgenic plants. All related primers are listed in Dataset 2.

### Phylogenetic analysis of rice varieties

The phylogeny assignment to the 110 rice varieties is based on SNP-resolved whole-genome data published previously [14]. Whole-genome SNP sequences were generated with plink v.2.0 [32]. Bootstrap resampling, a nucleic acid sequence distance matrix, and the neighbor-joining method were used to construct an unrooted tree and calculated with SEQBOOT, DNADIST, and NEIGHBOR available within phylip v.3.698 [33], respectively. Unclassified rice varieties in the RDP-II collection were assigned to the *indica* and *japonica* subgroups based on classified rice varieties and phylogenetic distances to them (Fig. S3). The resulting phylogenetic tree was visualized with the web tool iTOL (https://itol.embl.de/).

### Rice leaf metabolite extraction

Five-week-old plants cultivated under greenhouse conditions were harvested for extraction of leaf metabolites (10 replicates per genotype, each replicate included three plant leaves). Rice leaves were immediately frozen in liquid nitrogen and ground into fine powder with a mortar and pestle. Leaf metabolites were extracted from 0.5 g of each sample using 5 mL acetonitrile (ACN). The homogenates were shaken at 200 rpm/min for 10 min, followed by sonication at a frequency of 30 kHz for 30 min. After centrifugation at 19,000 g, 4 °C for 10 min, the supernatant was obtained and mixed with 30 mg GCB, and then subjected to centrifugation at 19,000 g, 4 °C for 5 min. The supernatant was filtered through a 0.22-μm filter before analysis with a LC–MS system.

### LC–MS conditions

The samples were separated by ultra-high–performance liquid chromatography (UPLC) on an UPLC BEH C-18 column (1.7 µm, 2.1 mm × 100 mm, Waters, USA); the column temperature was 30 °C. The flow rate was set at 0.3 mL/min, and the injection volume was 10 μL. For separation of LPA, SCPT, and FLA, the mobile phase consisted of chromatography-grade ACN (solvent A) and 0.5% (v/v) formic acid/H_2_O (solvent B). The gradient elution procedure was as follows: 0–1.0 min, 10% A/B (v/v); 1–1.5 min, linearly changed to 95% A/B (v/v); 1.5–3.5 min, A/B was maintained at 95% (v/v); 3.5–4.0 min, A/B linearly changed to 10% (v/v); and 4.0–5.0 min, A/B was maintained at 10%. For separation of HCA, CFA, and SPA, the mobile phase consisted of chromatography-grade ACN (solvent A) and 0.5% (v/v) ammonium formate/H_2_O (solvent C). The gradient elution procedure was as follows: 0–1.0 min, 95% A/C (v/v); 1–3 min, linearly changed to 40% A/C (v/v); 3–3.5 min, A/C was maintained at 40% (v/v); 3.5–4.0 min, A/C linearly changed to 95% (v/v); and 4.0–5.0 min, A/C was maintained at 95%.

In MS acquisition, the positive and negative mode of the ESI source as well as multiple reaction monitoring (MRM) mode were used for target metabolite analysis. Capillary voltage, source temperature, desolvation temperature, con gas flow, and desolvation gas flow of the instrument were set to 3.0 kV, 120 °C, 350 °C, 50 L/h, and 800 L/h, respectively. The optimized MRM parameters for the target metabolites are provided in Table S1. In order to avoid influences caused by fluctuations of the instrument, a random sequence was used for the analysis of all samples.

### Quantification of metabolites in rice leaves

Standard concentrations of the compounds were prepared at 0.001, 0.01, 0.05, 0.1, 0.5, and 1 mg/L. The standard preparations were mixed with 30 mg GCB, then shaken, centrifuged and filtered before LC–MS experiments. The standard curve was calculated with the linear regression method by using ggplot2. Recovery tests were performed by measuring the metabolite concentrations in 0.5 g rice leaves supplemented with 0.1 mL of the 100 mg/L standard solution for each target compound using the aforementioned method for extraction and detection. The correlation coefficients (*R*^2^) of the standard curves ranged from 0.9907 to 0.9998, and the mean recoveries of the target compounds ranged from 70.4 to 80.7% (Table S2).

A principal coordinate analysis (PCoA) and a permutational multivariate analysis of variance (PERMANOVA, 999 permutations) based on Bray–Curtis distances were performed using the R package vegan v.2.6–4 [34]. Comparative analysis of metabolite concentrations was performed using unpaired two-tailed Student’s *t*-test. Metabolite concentrations were defined as significantly different if the *p*-value was < 0.05. Correlation analyses with metabolite were conducted by using the R package GGally v.2.1.2 [35]. PCoA and metabolite concentration data were visualized with R package ggplot2 v.3.4.2 [36]. Scripts used for all analyses described here are available in the Code Availability section.

### Phyllosphere sample preparation, 16S rRNA gene fragment library generation, and sequencing

Five-week-old plants cultivated under greenhouse conditions were harvested for profiling of the phyllosphere microbiome (10 replicates per genotype, each replicate contained leaves from three plants). The new and second leaves, in order to avoid potential contaminations from soil, were collected from each plant. A total of 1 g leaf material from each rice variety was used to enrich the microbiota from the plant phyllosphere. The leaf samples for each replicate were transferred into a 50-mL plastic tube containing 20 mL sterile PBS buffer (0.02 M, PH = 7.0). The tube was placed in a shaker for 10 min set at 200 rpm/min and then sonicated for 5 min at a frequency of 30 kHZ at 4 °C. After collection of the suspensions, the leaves were treated with the same oscillation and sonication procedures twice to ensure that bacterial cells were thoroughly washed off from the leaf surface. The suspensions were pooled together and subjected to centrifugation (16,980 g, 10 min, 4 °C). After combining all suspensions, the samples were stored at − 80 °C before further use.

Total DNA was extracted using the MagPure Soil DNA LQ Kit (Magen, Shanghai, China) according to the manufacturer’s instructions. Quality and quantity of DNA was verified using a NanoDrop ND-1000 spectrophotometer (Thermo Fisher Scientific, USA) and agarose gel electrophoresis, respectively. Extracted DNA was diluted to a concentration of 1 ng/μL and stored at − 20 °C until further processing. PCR amplification of bacterial 16S rRNA gene fragments (V3-V4 regions) was performed using Takara Ex Taq (Takara, Beijing, China) and the primers 343 F (5’-TACGGRAGGCAGCAG-3’) and 798R (5’-AGGGTATCTAATCCT-3’). Amplicons were visualized using agarose gel electrophoresis and purified using Agencourt AMPure XP beads (Beckman Coulter, Pasadena, USA) twice. After purification, the DNA was quantified using Qubit dsDNA assay kit (Yeasen, Shanghai, China). Equal amounts of purified DNA were pooled for sequencing on the NovaSeq 6000 platform (Illumina Inc., USA) at Shanghai OEbiotech (Shanghai, China).

### Bioinformatic analysis for phyllosphere microbiome profiling

The 16S rRNA gene fragment sequences were processed using vsearch v.2.22.1 [37]. Paired-end reads were merged, low-quality sequences were filtered, and the primers were removed using “fastq_mergepairs,” “fastx_filter,” and “fastq_stripleft” commands in vsearch, respectively. After trimming, paired-end reads were dereplicated, clustered and denoised using “derep_fulllength,” “cluster_size,” and “uchime_ref” commands in vsearch, respectively. In the present study, a total of 86,083,925 paired-end raw data reads were obtained, with an average length of 250 bp. After filtering out low-quality, deduplicated, and chimeric reads, 60,353,250 high-quality reads were retained for rice microbiome analysis. The operational taxonomic unit (OTU) table was generated using “usearch_global” command in vsearch (Dataset 3). All OTUs were annotated using the Silva v138.1 reference database [38].

Alpha diversity analysis was carried out using EasyAmplicon script [39]. A principal coordinate analysis (PCoA) based on Bray–Curtis distances was performed using the R package vegan. Differences in beta diversity were accessed using a permutational analysis of variance (PERMANOVA, 999 permutations) available in the R package vegan. Analysis of differential species abundance and determination of significantly different abundant taxa was performed using the same methods as described for the metabolite analyses. Diversity and relative species abundance data were visualized by using R package ggplot2.

OTU sequences (relative abundance > 0.02%) were selected using fastx_getseqs command in usearch v.10.0.240 [40]. The selected sequences were aligned using MUSCLE v.3.8.1551 [41]. A phylogenetic tree was constructed and visualized using FastTree v.2.1.11 [42] and the web tool iTOL (https://itol.embl.de/), respectively. Scripts used for all analyses described here are available in the Code Availability section.

### Association analysis between leaf metabolites and the phyllosphere microbiome

Redundancy analysis (RDA) was performed using the R package vegan. Differences in the effect size of the microbiome were assessed using a permutation test (999 permutations) with the “envfit” command in the R package vegan. Correlations between metabolites and the microbiome were obtained using two-sided Pearson coefficients. RDA and correlation data were visualized by using R package ggplot2. Scripts used for all analyses described here are available in the Code Availability section.

### Association analysis between metabolites and phenylpropanoid biosynthesis pathway genes

The analysis of metabolite-gene associations is based on 110 C-RDP-II rice accessions represented by a SNP dataset [14] (filter removal MAF < 0.05). The SNPs of phenylpropanoid biosynthesis pathway genes were extracted from the C-RDP-II collection based on the Rice Annotation Project Database (RAPDB) [43]. We defined “traits” as mean concentrations of ferulic acid, l-phenylalanine, and caffeic acid in the leaves of the 110 rice varieties. Tassel5.0 (https://www.maizegenetics.net/tassel) and an analysis pipeline were set up on a Linux system to analyze the concentration “traits” individually (scripts are available in the Code Availability section). We used a mixed linear model (MLM) that integrated the kinship matrix (K) with population structure (Q). Metabolite-associated SNPs were screened for maximum significantly associated SNPs using a *p-*value threshold (*p* < 0.05, Dataset 4).

The association level of each gene was defined through comparison of the lowest *p-*value of metabolite-associated SNPs among each relevant gene (Dataset 5). The genes of the top 10 associations were used to link the microbiome base on previous research [14]. The genes that were both associated with metabolite and the microbiome were used to analyze their haplotype. Comparative analysis of metabolite concentrations and bacterial abundance were performed using unpaired two-tailed Student’s *t* test and visualized by using R package ggplot2.

### Effect of ferulic acid on leaf bacterial growth

Members of Pseudomonadales and Burkholderiales were isolated from rice plants cultivated under the aforementioned greenhouse conditions and identified by Sanger sequencing with 27 F and 1492R primers (Dataset 6). Individual bacterial strains from the culture collection were picked from R2A agar plates and grown overnight in R2A medium on a shaker at 180 rpm/min, 28 °C. Overnight cultures were used to collect bacteria with centrifugation at 600 g, 4 °C for 10 min. The collected bacteria were diluted to OD_600_ = 0.01 using R2A medium (supplemented with 0, 0.2, 0.5, or 1 mM FLA). Then, 200 µL of each microbial suspension was aliquoted into clear 96-well flat-bottom, polystyrene tissue culture plates. The plates were incubated at 28 °C on an orbital shaking platform at 80 rpm/min. The optical density (OD_600_) was measured every 2 h at 600 nm and monitored over 24 h. All isolates were tested in triplicates with two technical repeats.

## Results

### Phenylpropanoid concentrations in rice leaves differ between genotypes

**Fig. 1:**
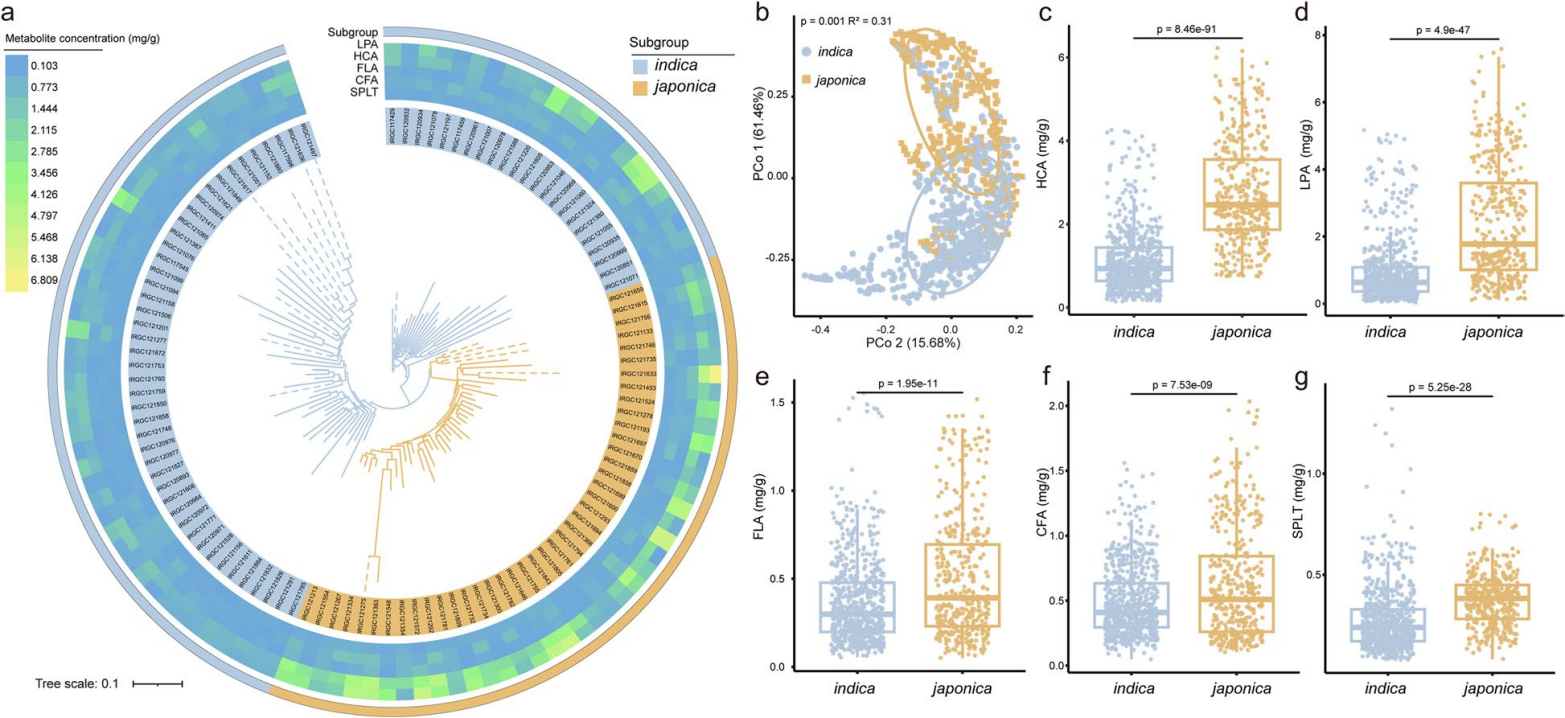
Metabolite profiles of 110 rice varieties. **a** Phylogenetic tree and leaf phenylpropanoid concentrations among 110 rice varieties (Dataset 7). The inner part represents the phylogeny of 110 rice varieties based on genomic-scale SNP differences. Dashed lines and solid lines indicate unclassified and classified rice varieties within RDP-II, respectively. The heatmap represents the phenylpropanoid concentrations in the rice leaves. The outer ring represents *indica* and *japonica* subgroups based on classified rice varieties and phylogenetic distance. **b** Unconstrained PCoA based on Bray–Curtis distances showing leaf phenylpropanoid separation for *japonica* and *indica* rice varieties (*p* = 0.001, *R*^2^ = 0.31, *p*-values were calculated through one-way PERMANOVA). Ellipses cover 60% of the data for each group. Cyan and orange colors represent *indica* and *japonica* rice samples, respectively. **c**–**g** Leaf concentrations of HCA (4-hydroxycinnamic acid, **c**), LPA (l-phenylalanine, **d**), FLA (ferulic acid, **e**), CFA (caffeic acid, **f**), and SPLT (scopoletin, **g**) between *japonica* and *indica* rice varieties. The horizontal bars within boxes represent medians. The tops and bottoms of boxes represent the 75th and 25th percentiles, respectively. The upper and lower whiskers extend to data no more than 1.5 × the interquartile range from the upper edge and lower edge of the box, respectively. The *p*-value was calculated with an unpaired two-tailed Student’s *t* test. In this figure, *indica* (*n* = 68) and *japonica* (*n* = 42) rice samples were used with ten replications for each genotype

A genome-wide association analysis (GWAS) from a previous study has provided indications for the involvement of specific lignin precursors in shaping the plant microbiome [14]. To further explore why genotype-specific microbiome variation occurs across rice genotypes, we first quantified five lignin precursors in rice leaves, including 4-hydroxycinnamic acid, ferulic acid, caffeic acid, sinapic acid, and l-phenylalanine from the phenylpropanoid biosynthesis pathway and one phenylpropanoid derivative, the coumarin “scopoletin.” The latter was previously reported to play an important role in assembling a plant health-promoting microbiome [20]. All conducted experiments are based on 68 *indica* and 42 *japonica* accessions from the Rice Diversity Panel II core collection (C-RDP-II) (Dataset 3). The targeted metabolite quantifications indicated that the concentrations of all six compounds significantly differed between *indica* and *japonica* accessions, albeit to varying degrees (Fig. 1a and Dataset 7). An unconstrained principal coordinate analysis (PCoA) with metabolite profiles showed that the first principal coordinate PCo1 accounted for 61.46% of total variance between *indica* and *japonica* accessions (Fig. 1b; *p* = 0.001, *R*^2^ = 0.31, PERMANOVA). This result is indicative of substantial heterogeneity in the natural variation of phenylpropanoid concentrations. Further analysis revealed that 4-hydroxycinnamic acid (Fig. 1c; the concentration in *japonica* accessions is 2.42-fold higher than in *indica* accessions), l-phenylalanine (Fig. 1d; 2.52-fold higher in *japonica* accessions), ferulic acid (Fig. 1e; 1.36-fold higher in *japonica* accessions), caffeic acid (Fig. 1f; 1.28-fold higher in *japonica* accessions), and scopoletin (Fig. 1g; 1.35-fold higher in *japonica* accessions) substantially differed between the two rice subgroups (Table S3). In contrast, sinapic acid was present in a significantly lower concentration in *japonica* accessions; however, the difference was low in comparison to the other compounds (Table S3). Further analysis showed that 4-hydroxycinnamic acid and l-phenylalanine concentrations were positively correlated with each other (Fig. S4; *p* < 0.0001, *R* = 0.851, *p*-value was calculated through two-sided Pearson coefficient). This is likely because l-phenylalanine serves as the main substrate for the enzyme phenylalanine ammonia-lyase (PAL) that catalyzes the biosynthesis of 4-hydroxycinnamic acid [44]. Collectively, our data suggested that phenylpropanoid concentrations substantially differ among rice genotypes, which prompted us to further investigate their potential role in the assembly of the phyllosphere microbiome.

### Microbial communities show substantial variation between rice genotypes

**Fig. 2:**
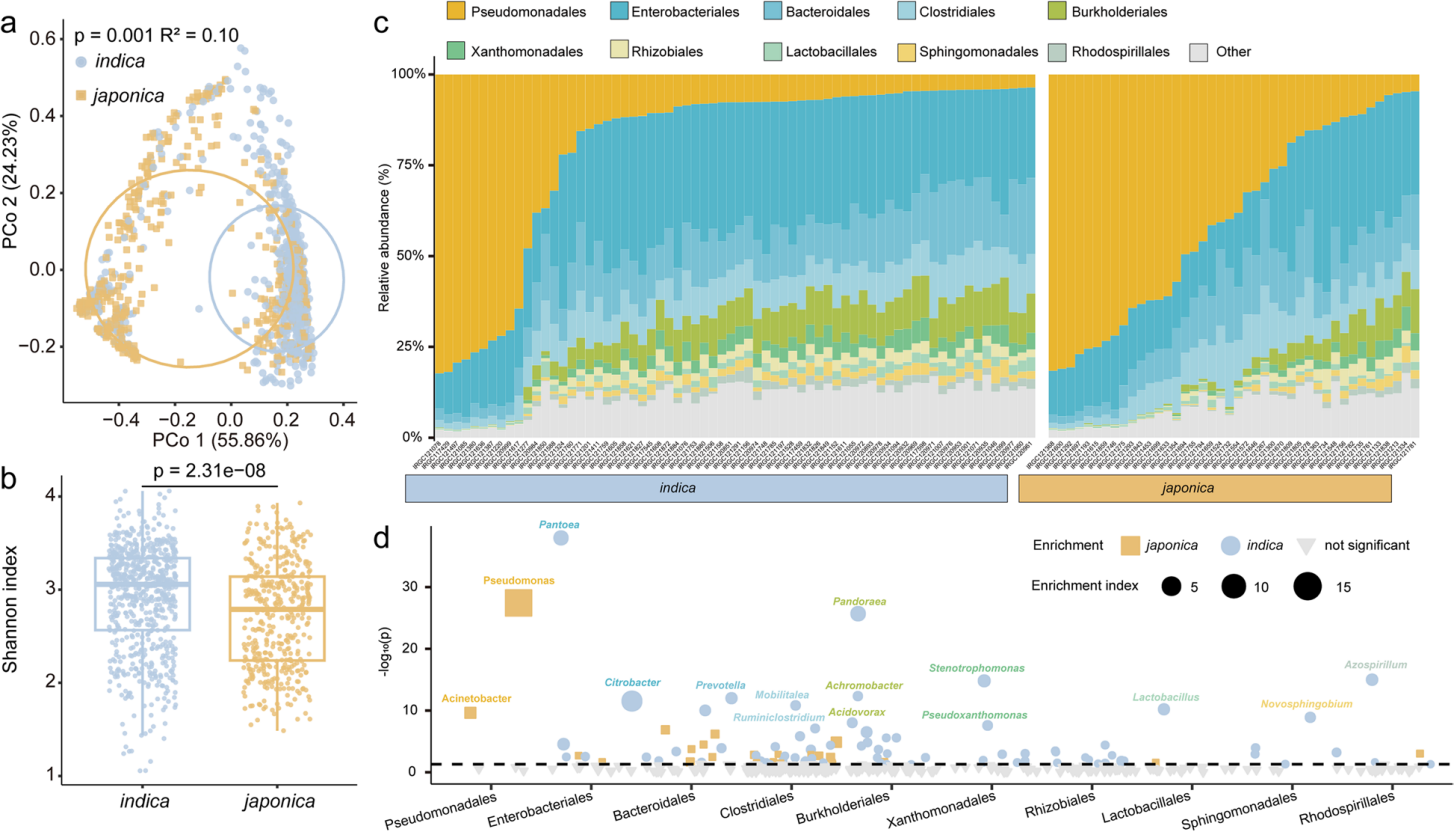
Phyllosphere microbiome profiles of 110 rice varieties. **a** Unconstrained PCoA (for principal coordinates PCo1 and PCo2) based on Bray–Curtis distances showing bacterial community clustering of *japonica* and *indica* rice varieties (*p* = 0.001, *R*^2^ = 0.10, *p* value was calculated through one-way PERMANOVA). Ellipses cover 60% of the data for each rice subgroups. **b** Shannon index for phyllosphere bacterial communities of *indica* and *japonica* varieties. Box plot percentiles are the same as in Fig. 1c. The *p* value was calculated with an unpaired two-tailed Student’s *t* test. **c** Order-level distribution of bacteria in the phyllosphere microbiomes. Ten replications were used for each plant genotype. **d** Comparison of relative abundances of bacterial genera among the top 10 orders between *indica* and *japonica* varieties. Squares and circles represent genera with significant relative abundance differences in *japonica* and *indica* rice varieties, respectively. The different sizes (enrichment index) of the shapes represent the difference between the mean values of relative abundance in *japonica* and *indica* rice varieties. Different colors of text represent distinct bacterial orders. The *p* value was calculated with an unpaired two-tailed Student’s *t* test. In this figure, *indica* (*n* = 68) and *japonica* (*n* = 42) rice samples were included with ten replications for each genotype

To assess the extent of microbiome variation between different rice genotypes under controlled conditions, we conducted 16S rRNA gene fragment amplicon sequencing. This allowed us to profile the phyllosphere microbiomes of the same rice accessions that were subjected to the phenylpropanoid quantifications described above. When the microbiomes were comparatively assessed, *indica* and *japonica* accessions formed two distinct clusters, as indicated by unconstrained principal coordinate analysis (PCoA) of Bray–Curtis distances (Fig. 2a; *p* = 0.001, *R*^2^ = 0.10, PERMANOVA). This observation suggested that *indica* and *japonica* accessions have distinct phyllosphere microbiome compositions and structures. Furthermore, the Shannon index and richness (α-diversity) were found to be significantly higher in *indica* as compared to *japonica* accessions, suggesting that the *indica* phyllosphere microbiome was more diverse (Fig. 2b and Fig. S5). Detailed analysis of bacterial operational taxonomic units (OTUs) also showed significant differences between *indica* and *japonica* accessions (Fig. S6 and Dataset 3). We observed that the phyllosphere microbiome was dominated by the bacterial orders Pseudomonadales, Enterobacterales, Bacteroidales, Clostridiales, Burkholderiales, Xanthomonadales, Rhizobiales, Lactobacillales, Sphingomonadales, and Rhodospirillales (Fig. 2c). Compared to *japonica*, *indica* accessions had a higher relative abundance of Enterobacterales, Burkholderiales, Xanthomonadales, Rhizobiales, Lactobacillales, Sphingomonadales, and Rhodospirillales, while *japonica* accessions had a higher relative abundance of Pseudomonadales (Dataset 3; unpaired two-tailed Student’s *t* test, *p* < 0.05). At the genus level, *Pseudomonas* and *Acinetobacter* were significantly enriched in the phyllosphere of *japonica* accessions (Fig. 2d and Dataset 3; unpaired two-tailed Student’s *t* test, *p* < 1.0E − 7). In contrast, *Pantoea*, *Citrobacter, Prevotella*, *Mobilitalea*, *Ruminiclostridium*, *Pandoraea*, *Achromobacter*, *Acidovorax*, *Stenotrophomonas*, *Pseudoxanthomonas*, *Lactobacillus*, *Novosphingobium,* and *Azospirillum* were significantly enriched in the *indica* accessions (Fig. 2d and Dataset 3; unpaired two-tailed Student’s *t* test, *p* < 1.0E − 7).

### Phenylpropanoids explain rice phyllosphere microbiome variation among genotypes

**Fig. 3:**
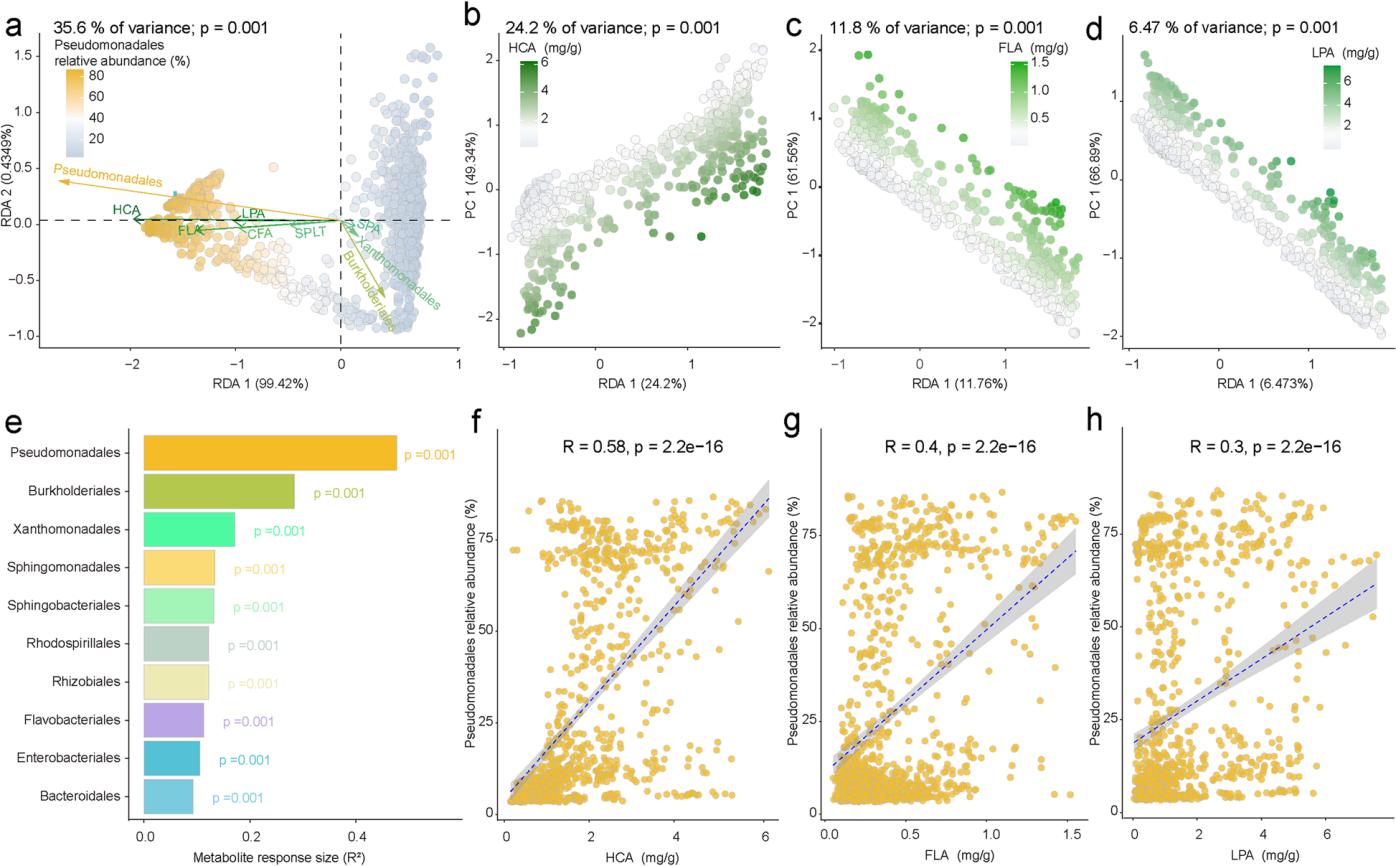
Metabolite-driven phyllosphere microbiome assembly. **a** RDA (redundancy analysis) was used to link the phyllosphere microbiome to differences in leaf phenylpropanoid concentrations (35.6% of the total variance of phyllosphere microbiome was explained by the leaf metabolites, *p* = 0.001, *p* value was calculated through one-way PERMANOVA). Regular arrows indicate different metabolites; triangle arrows indicate metabolite-responsive members of the core microbiome; different colors indicate differences in relative abundance of Pseudomonadales. **b–d** Phyllosphere microbiome variance explained by HCA (4-hydroxycinnamic acid, **b** explained 24.2% of total variance, *p* = 0.001), FLA (ferulic acid, **c** 11.8% of total variance, *p* = 0.001), and LPA (l-phenylalanine, **d** 6.47% of total variance, *p* = 0.001), respectively. The *p* value was calculated through one-way PERMANOVA; different colors indicate the content of metabolite. **e** Top 10 metabolite-responsive bacterial orders in the rice phyllosphere. The *p* value was calculated through permutation tests (999 permutations). **f–h** Correlation between relative abundance of Pseudomonadales and HCA concentrations (**f**), FLA (**g**), and LPA (**h**) in rice leaves, respectively. The two-sided Pearson coefficient *R* and *p* values were calculated using ggplot2; the gray area shows the 95% confidence interval of the regression line (blue dashed line). In this figure, 110 rice varieties with ten replications for each genotype are shown

In order to specifically address potential correlations between host metabolites on microbiome variation, we further carried out a redundancy analysis (RDA) to search for potential correlations between the six metabolites (4-hydroxycinnamic acid, l-phenylalanine, ferulic acid, caffeic acid, scopoletin, and sinapic acid) and the phyllosphere microbiome. We found that the concentrations of these compounds explained 35.6% of total phyllosphere microbiome variation (Fig. 3a; *p* = 0.001, PERMANOVA), indicating a strong correlation between phenylpropanoids and rice phyllosphere microbiome assembly. Moreover, we found that the concentrations of 4-hydroxycinnamic acid, ferulic acid, l-phenylalanine, caffeic acid, and scopoletin were correlated with rice varieties that had a higher relative abundance of Pseudomonadales. In contrast, increased sinapic acid content was correlated with rice varieties that had a lower relative abundance of Pseudomonadales (Fig. 3a). Further, we used RDA to explore the effects of specific phenylpropanoids on the phyllosphere microbiome. Here, 4-hydroxycinnamic acid, ferulic acid, l-phenylalanine, caffeic acid, scopoletin, and sinapic acid explained 24.2% (Fig. 3b; *p* = 0.001), 11.8% (Fig. 3c; *p* = 0.001), 6.47% (Fig. 3d; *p* = 0.001), 5.85% (Fig. S7a; *p* = 0.001), 1.41% (Fig. S7b; *p* = 0.001), and 0.13% (Fig. S7c, *p* = 0.22) of the total phyllosphere microbiome variation, respectively (*p* values were calculated through PERMANOVA). These results indicate that different phenylpropanoids affect phyllosphere microbiome variation to differing extents and that 4-hydroxycinnamic acid is the main driver of microbiome assembly among the six metabolites. Additionally, RDA indicated that the top 10 bacterial orders (out of 47 orders in total) that significantly responded to the metabolites were Pseudomonadales (*R*^2^ = 0.477), Burkholderiales (*R*^2^ = 0.285), Xanthomonadales (*R*^2^ = 0.172), Sphingomonadales (*R*^2^ = 0.134), Sphingobacteriales (*R*^2^ = 0.132), Rhodospirillales (*R*^2^ = 0.122), Rhizobiales (*R*^2^ = 0.122), Flavobacteriales (*R*^2^ = 0.113), Enterobacterales (*R*^2^ = 0.106), and Bacteroidales (*R*^2^ = 0.093) (Fig. 3e and Dataset 3; *p* < 0.05, *p* values were calculated through permutation tests). A correlation analysis was used to further confirm the relationship between the bacterial order Pseudomonadales and different metabolites. This was guided by the RDA results, which indicated the highest *R*^2^ value for Pseudomonadales. The relative abundance of Pseudomonadales showed a positive correlation with the concentrations of 4-hydroxycinnamic acid (Fig. 3f; *R* = 0.58), ferulic acid (Fig. 3g; *R* = 0.4), L-phenylalanine (Fig. 3h; *R* = 0.3), caffeic acid (Fig. S7d; *R* = 0.28), and scopoletin (Fig. S7e; *R* = 0.14), respectively (*p* < 0.0001, *p* value was calculated through two-sided Pearson coefficient). In addition, the concentration of 4-hydroxycinnamic acid showed a significantly negative correlation with the relative abundances of Burkholderiales (*R* = − 0.48), Xanthomonadales (*R* = − 0.38), Sphingomonadales (*R* = − 0.31), and Sphingobacteriales (*R* = − 0.34), respectively (Fig. S8; *p* < 0.0001, *p* value was calculated through two-sided Pearson coefficient). Taken together, our data provided clear evidence that phenylpropanoids are associated with phyllosphere microbiome variation among different rice genotypes.

### Identification of phenylpropanoid biosynthesis pathway genes associated with metabolite-driven microbiome assembly

Previously, we have shown that OsPAL02, which is required for the biosynthesis of 4-hydroxycinnamic acid, is also linked to the enrichment of Pseudomonadales and inhibition of Xanthomonadales on rice leaves [14]. To further explore the potential presence of *M* genes in the phenylpropanoid biosynthesis pathway, we first conducted association analyses between single-nucleotide polymorphisms (SNPs) and the concentrations of ferulic acid, l-phenylalanine, and caffeic acid. This analysis resulted in the identification of 113, 144, and 136 genes with SNPs significantly associated with the concentrations of ferulic acid, l-phenylalanine, and caffeic acid, respectively (Dataset 4; *p* < 0.05). We selected 10 genes with the lowest *p* values from the association analysis between their SNPs and concentrations of ferulic acid, l-phenylalanine, and caffeic acid (Dataset 4) and comparatively assessed them with genes that we have previously discovered to be significantly associated with the relative abundances of specific microbial taxa [14]. We found that the genes *OsC4H2* (Os05g25640), *OsPAL06* (Os02g41680), and *prx49* (Os03g36560), which are associated with ferulic acid concentration, were also associated with the relative abundance of Burkholderiales. The gene *prx109* (Os07g47990), which is also associated with ferulic acid concentration, was associated with the relative abundance of Pseudomonadales. In addition, the two genes *prx8* (Os01g18930) and *prx11* (Os01g19020), which are associated with the concentrations of l-phenylalanine, were associated with the relative abundance of Xanthomonadales. The gene *prx112* (Os07g48030), which is associated with the concentrations of caffeic acid, was associated with the relative abundance of Pseudomonadales.

**Fig. 4:**
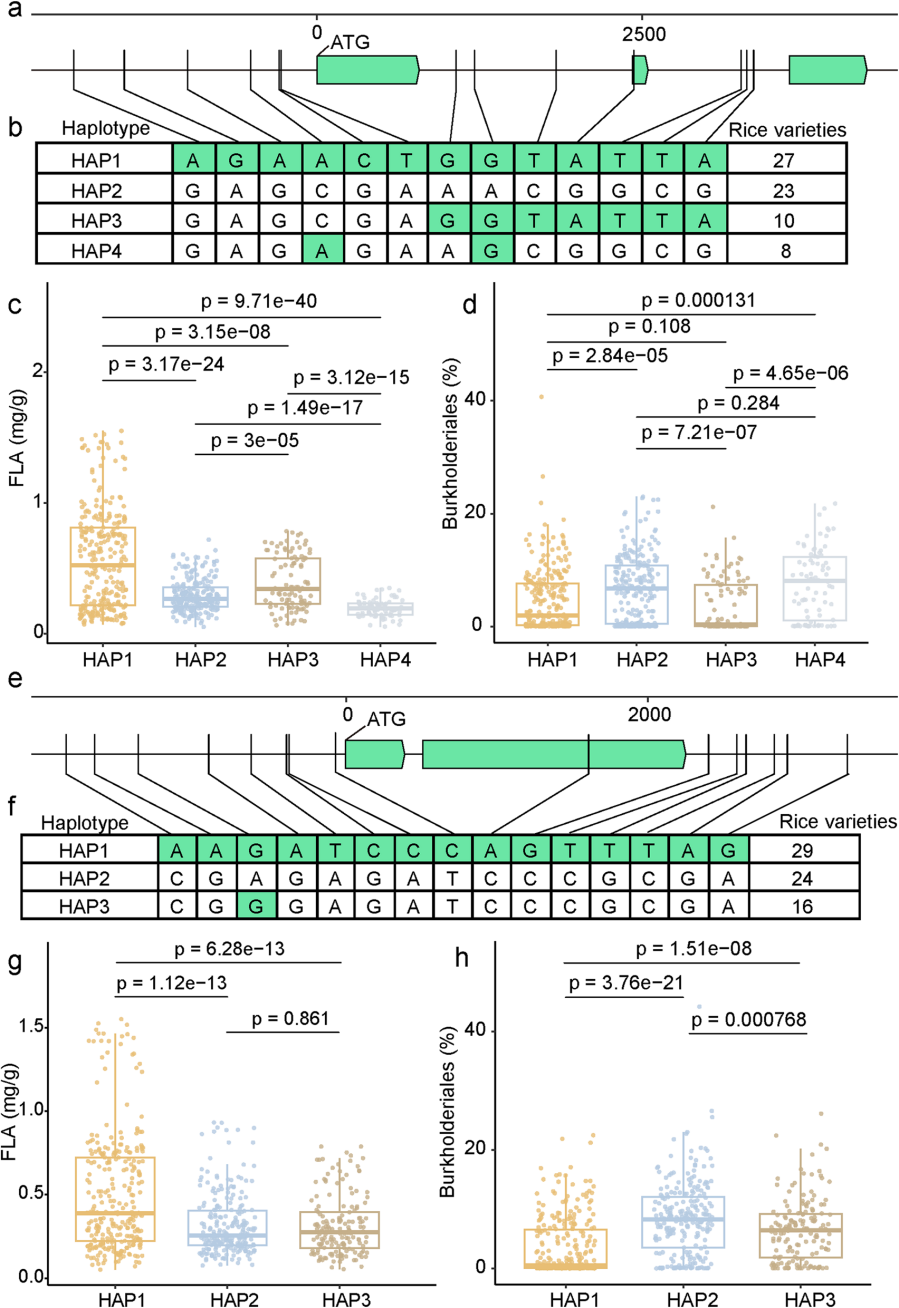
*OsC4H2* and *OsPAL06* variants are linked to FLA concentrations and Burkholderiales abundance.** a** Gene structure of *OsC4H2*. Bar arrows indicate the protein-coding region. Vertical lines indicate SNP loci. **b** Haplotype analysis of *OsC4H2*. Nucleotide differences are indicated in green. HAP1/2/3/4 indicate haplotypes 1/2/3/4. Leaf concentrations of FLA (ferulic acid, **c**) and abundance of Burkholderiales (**d**) between different *OsC4H2* haplotypes of the 110 rice varieties. HAP1 (*n* = 27), HAP2 (*n* = 23), HAP3 (*n* = 10), and HAP4 (*n* = 8) rice samples were used with ten replications for each genotype.** e** Gene structure of *OsPAL06*. Bar arrows indicate the protein-coding region. Vertical lines indicate SNP loci. **f** Haplotype analysis of *OsPAL06*. Nucleotide differences are indicated in green. HAP1/2/3 indicate haplotypes 1/2/3. Leaf concentrations of FLA (ferulic acid, **g**) and abundance of Burkholderiales (**h**) between different *OsPAL06* haplotypes of the 110 rice varieties. Calculations for HAP1 (*n* = 29), HAP2 (*n* = 24), and HAP3 (*n* = 16) were conducted with ten replications for each genotype. In this figure, box plot percentiles are the same as in Fig. 1c. *P* values were calculated with an unpaired two-tailed Student’s *t* test

We decided to further verify genes responsible for metabolite-driven microbiome assembly and focused on sequence variations of *OsC4H2* and *OsPAL06* across 110 rice varieties. This analysis identified four major haplotypes for *OsC4H2* and three major haplotypes for *OsPAL06* (Fig. 4). Rice varieties carrying *OsC4H2* haplotypes 1 and 3 (Fig. 4a and 4b) showed significantly higher ferulic acid concentrations compared with varieties carrying *OsC4H2* haplotypes 2 and 4 (Fig. 4c; unpaired two-tailed Student’s *t* test, *p* < 0.001). The relative abundance of Burkholderiales in *OsC4H2* haplotype 1 and 3 varieties was significantly lower than in *OsC4H2* haplotype 2 and 4 varieties (Fig. 4d; unpaired two-tailed Student’s *t* test, *p* < 0.001). Sequence comparison of the *OsC4H2* haplotypes revealed six nucleotide variations in the promoter region between *OsC4H2* haplotype 1 and the other haplotypes, and one single nucleotide variation in the coding region between *OsC4H2* haplotypes 1 and 3 and haplotypes 2 and 4 (Fig. 4a, b). Similarly, rice varieties carrying *OsPAL06* haplotype 1 (Fig. 4e, f) were linked to significantly higher ferulic acid concentrations compared with the varieties carrying *OsPAL06* haplotypes 2 and 3 (Fig. 4g; unpaired two-tailed Student’s *t* test, *p* < 1.0E − 7). The relative abundance of Burkholderiales in *OsPAL06* haplotype 1 was significantly lower than in *OsPAL06* haplotypes 2 and 3 (Fig. 4h; unpaired two-tailed Student’s *t* test, *p* < 1.0E − 7). Sequence comparison of the *OsPAL06* haplotypes revealed eight nucleotide variations in the promoter region between *OsPAL06* haplotype 1 and the other haplotypes, and one single nucleotide variation in the coding region between *OsPAL06* haplotype 1 and haplotypes 2 and 3 (Fig. 4e, f). *OsC4H2* encodes a protein with trans-cinnamic acid 4-hydroxylase activity, which catalyzes the conversion of trans-cinnamate and cinnamoyl-CoA into 4-hydroxycinnamic acid and *p*-coumaroyl-CoA, respectively [45]. *OsPAL06* encodes a protein with phenylalanine ammonia-lyase activity, which catalyzes l-phenylalanine conversion into trans-cinnamate [46]. The catalytic products of both proteins are precursors required for the biosynthesis of ferulic acid. Therefore, we hypothesize that the nucleotide variations of *OsC4H2* and *OsPAL06* are linked to different expression levels or enzymic activities of their encoded proteins across rice varieties, leading to variations in ferulic acid concentrations in rice.

#### Validation of ferulic acid’s activity in shaping the phyllosphere microbiome

**Fig. 5:**
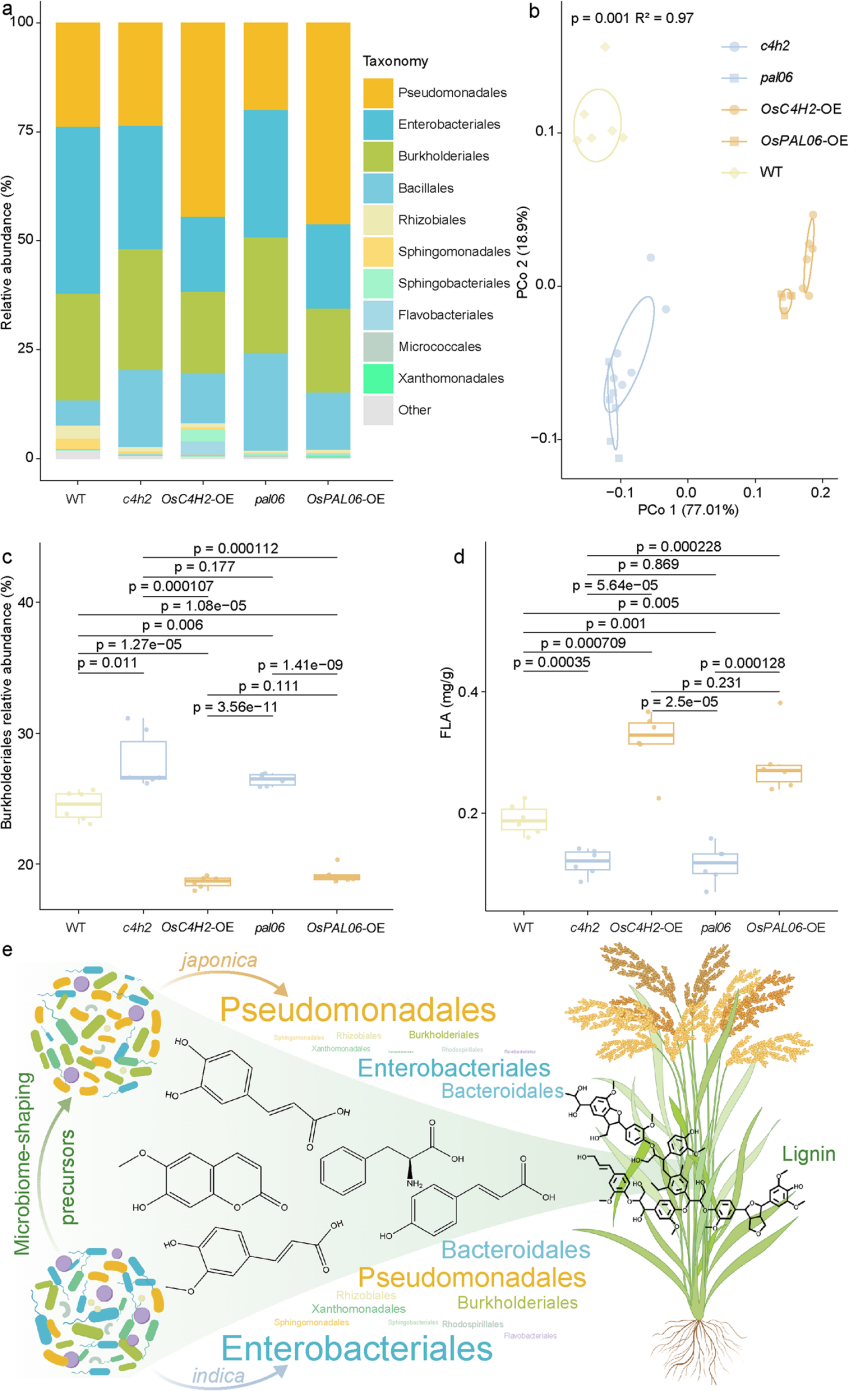
*M* genes regulate phyllosphere microbiome and FLA concentrations. **a** Bacterial communities at order level in the phyllosphere of WT (wild-type of ZH11 rice variety), *c4h2* (*OsC4H2*-knockout), *OsC4H2*-OE (*OsC4H2*-overexpression), *pal06* (*OsPAL06*-knockout), and *OsPAL06*-OE (*OsPAL06*-overexpression) plants. Six replications were used for each plant genotype. **b** Unconstrained PCoA (for principal coordinates PCo1 and PCo2) based on Bray–Curtis distances showing bacterial community clustering of WT, *c4h2*, *OsC4H2*-OE, *pal06*, and *OsPAL06*-OE plants (*p* = 0.001, *R*^2^ = 0.10, *p* value was calculated through one-way PERMANOVA). Ellipses cover 60% of the data for each rice subgroup. **c**,** d** Comparison of relative abundances of Burkholderiales (**c**) and concentrations of FLA (ferulic acid, **d**) between WT, *c4h2*, *OsC4H2*-OE, *pal06*, and *OsPAL06*-OE plants. The numbers of replicated samples are as follows: WT (*n* = 6), *c4h2*, (*n* = 6), *OsC4H2*-OE (*n* = 6), *pal06* (*n* = 6), and *OsPAL06*-OE (*n* = 6). Box plot percentiles are the same as in Fig. 1c. *P*-values were calculated with an unpaired two-tailed Student’s *t* test. **e** Schematic visualization of the discovered effects of specific lignin monomers that are under the control of *M* genes on the phyllosphere microbiome of rice (created with Figdraw). Concentration differences of specific phenylpropanoids shape the microbiomes of the *indica* and *japonica* subgroups

To validate the gene functions of *OsC4H2* and *OsPAL06* with respect to their microbiome-shaping effects, we successfully constructed their knockout mutants in rice (*c4h2*, *pal06*) and overexpression lines (*OsC4H2-*OE, *OsPAL06-*OE), using the wild-type (WT) rice variety ZH11 (*japonica* group). Microbiome profiling identified Pseudomonadales, Enterobacterales, Burkholderiales, Bacillales, Rhizobiales, Sphingomonadales, Sphingobacteriales, Flavobacteriales, Micrococcales, and Xanthomonadales as the dominant taxa in the phyllosphere of *c4h2*, *pal06*, *OsC4H2-*OE, *OsPAL06-*OE, and WT plants (Fig. 5a). Principal coordinates analysis (PCoA) showed significant separation among the phyllosphere microbiome of the *c4h2*, *pal06*, *OsC4H2-*OE, *OsPAL06-*OE, and WT plants, confirming the implications of *OsC4H2* and *OsPAL06* in shaping the phyllosphere microbiome (Fig. 5b; *p* = 0.001, PERMANOVA). Differential abundance analysis revealed that the relative abundance of Burkholderiales in *c4h2* and *pal06* was significantly higher compared to WT plants, whereas it was significantly lower in the *OsC4H2-*OE and *OsPAL06-*OE lines (Fig. 5c; unpaired two-tailed Student’s *t* test, *p* < 0.05). Moreover, the relative abundance of Pseudomonadales in *pal06* was significantly lower than in WT plants (*p* < 0.05), but no significant difference between *c4h2* and the WT plants was observed (*p* = 0.851). In *OsC4H2-*OE and *OsPAL06-*OE plants, Pseudomonadales showed a significantly higher relative abundance than in WT plants (Fig. S9a; unpaired two-tailed Student’s t test, *p* < 0.05). Overall, the results indicated that both *OsC4H2* and *OsPAL06* are *M* genes that negatively regulate the enrichment of Burkholderiales and positively regulate the enrichment of Pseudomonadales in the rice phyllosphere.

To confirm that *OsC4H2* and *OsPAL06* shape the rice phyllosphere microbiome through selective effects on specific microbial taxa exerted by metabolites, we quantified concentrations of ferulic acid and 4-hydroxycinnamic acid in leaf tissues of *c4h2*, *pal06*, *OsC4H2-*OE, *OsPAL06-*OE, and WT plants. Ferulic acid concentrations in both *c4h2* and *pal06* were significantly lower than in WT plants and significantly higher in *OsC4H2-*OE and *OsPAL06-*OE when compared with WT plants (Fig. 5d; unpaired two-tailed Student’s *t* test, *p* < 0.05). In addition, 4-hydroxycinnamic acid concentrations in *pal06* were significantly lower than in WT plants (*p* < 0.05), but no significant difference was observed between *c4h2* and WT plants (*p* = 0.763); however, 4-hydroxycinnamic acid concentrations were significantly increased in *OsC4H2-*OE and *OsPAL06-*OE when compared with WT plants (Fig. S9b; unpaired two-tailed Student’s *t* test, *p* < 0.05). The results confirmed the role of *OsC4H2* and *OsPAL06* in both increased ferulic acid and increased 4-hydroxycinnamic acid biosynthesis. The relative abundance of Burkholderiales showed a negative correlation with the concentrations of ferulic acid (Fig. S9c; *R* = − 0.87) and 4-hydroxycinnamic acid (Fig. S9d; *R* = − 0.77) in *c4h2*, *pal06*, *OsC4H2-*OE, *OsPAL06-*OE, and WT plants (*p* < 0.0001, *p* value was calculated through two-sided Pearson coefficient). In addition, we conducted in vitro assays to test the effects of ferulic acid on 10 Burkholderiales and 10 Pseudomonadales strains isolated from the rice phyllosphere. When the ferulic acid concentration in liquid cultures reached 1 mM, the growth of all Burkholderiales strains was substantially inhibited. In contrast, either no or only weak inhibition was observed on Pseudomonadales strains (Fig. S10). This result provides an explanation for phyllosphere microbiome assembly discrepancies between *c4h2*, *pal06*, *OsC4H2*-OE, *OsPAL06*-OE, and WT plants and indicates a key regulatory role of ferulic acid. Taken together, our results suggest that *OsC4H2* and *OsPAL06* are M genes and shape the rice microbiome through the regulation of ferulic acid and 4-hydroxycinnamic acid biosynthesis.

## Discussion

Plants produce various metabolites that can affect microbiome assembly. This can be used to alleviate biotic and abiotic stresses or to recruit specific microbial taxa assisting nutrient uptake [47]. Prominent examples are cucurbitacins [48], coumarins [49], purines [6], and 4-hydroxycinnamic acid [14], all of which have been shown to alter the microbiome. In this study, we show that concentrations of phenylpropanoids, including l-phenylalanine, 4-hydroxycinnamic acid, ferulic acid, caffeic acid, and the phenylpropanoid derivative scopoletin, can substantially differ in rice leaves among rice genotypes. Complementary microbiome profiling indicated that these metabolites shape phyllosphere microbiome assembly through regulating the abundance of the predominant bacterial orders Pseudomonadales, Burkholderiales, and Xanthomonadales. Phenylpropanoids are ubiquitous in plant tissues and fulfill various essential roles [27]. They are not only involved in plant responses toward biotic and abiotic stresses but also precursor molecules that are required for the biosynthesis of structural polymers like lignin. In addition, the biosynthesis of specific plant defense compounds such as phenolic phytoalexins, tannins, and phytohormone salicylic acid requires phenylpropanoids as precursors [27]. Adding to these known roles, we found that plant phenylpropanoids play a central role in phyllosphere microbiome assembly. Our findings reveal a so far largely unknown function of these monomers that might be essential for shaping a functional phyllosphere microbiome (Fig. 5e). The involvement of phenylpropanoids in microbiome assembly also suggests that they are key elements plants rely on to shape their phenotype.

By combining ferulic acid quantification and phyllosphere microbiome profiling with genetically modified rice plants targeted at genes *OsC4H2* and *OsPAL06*, we uncovered the potential regulatory role of ferulic acid on microbiome assembly. Overexpression of the rice genes *OsC4H2* or *OsPAL06* led to increased concentrations of ferulic acid in leaf tissues and consequently enriched Pseudomonadales while suppressing Burkholderiales. The *OsC4H2* and *OsPAL06* knockout mutants exhibited contrary phenotypes to their respective overexpression lines, including decreased concentrations of ferulic acid and enriched Burkholderiales. These observations are also reflected in naturally occurring haplotypes, in which *OsC4H2* and *OsPAL06* encode enzymes that likely differ in their enzymic activities. Bacterial members from these two orders are ubiquitous plant-associated inhabitants and can be linked to plant health and disease. For example, *Pseudomonas* spp. are key players in disease protection due to their antimicrobial compounds and potential for plant defense induction [5]. *Burkholderia glumae* and *Burkholderia plantarii* are globally widespread, seed-borne, and toxin-producing pathogens in cereal crops, causing substantial damage to crop yield and quality [50]. In rice seeds, the prevalence of *Sphingomonas melonis* can lead to disease resistance against the seed-borne pathogen *Burkholderia plantarii* [50]. This indicates that suppression of *Burkholderia* pathogens could be achieved by modulating the seed-endophytic microbiota. The discovery of microbiome-shaping effects via *OsC4H2* and *OsPAL06*, and the corresponding metabolite ferulic acid, could provide the basis for new approaches to control *Burkholderia* pathogens. Interestingly, some *Burkholderia* isolates were reported to produce antifungal compounds and induce systemic resistance against pathogens, contributing to crop growth promotion and disease resistance [51]. Plant genes in the phenylpropanoid biosynthesis pathway are known to mediate broad-spectrum resistance against pathogens and herbivores by regulating the biosynthesis of the immunity-related signal molecule salicylic acid and the accumulation of lignin [46, 52]. Adding to those functions, our study revealed microbiome-shaping functions of *OsPAL06* and *OsC4H2* and extended our knowledge of how plants shape their microbiome to establish adaptive traits. We found that rice plants have distinct haplotypes of *OsPAL06* and *OsC4H2*, which were associated with differing ferulic acid concentrations and reduced abundance of Burkholderiales. In vitro assays indicated that ferulic acid exerted substantial inhibition on the growth of Burkholderiales strains (Fig. S10), further reinforcing this observation. A recent study found that the endophytic fugus *Acrocalymma vagum* releases coumaric and trans-ferulic acids in the rice holobiont to attract beneficial microbes and to suppress the potentially pathogenic *Burkholderia* spp. [53]. Overall, our results show that metabolite-driven enrichment or suppression of certain taxa in plants are the outcomes of *M* gene functioning which diverged in distinct genotypes.

It is increasingly noticed that many plants’ adaptive traits rely on specific microbiome structures [54]. Nitrogen use efficiency can largely depend on the enrichment of specific bacteria in the rhizosphere [4], and genotype-specific disease suppression and stress tolerance can be associated with microbiome composition [20, 55]. This highlights why fine-tuning microbiome assembly through host genetic control is of great importance to the plant’s environmental adaptability.

Host genes that shape the microbiome are known as Microbiome genes, or *M* genes in short, and will likely play a key role in future microbiome engineering approaches [14, 56, 57]. *M* genes are likely involved in many commonly observed phenomena related to the plant microbiome, like seasonal variations in the phyllosphere [58]. Interestingly, also in the human gut microbiome, the presence of specific bacterial genes associated with *N*-acetylgalactosamine utilization is significantly influenced by human genotypes [59].

## Conclusion

In plants, the underlying reasons for genotype specificity of the microbiome were mostly elusive so far. We could show that a rather small set of six host metabolites that likely are under the control of *M* genes can explain more than one-third of the phyllosphere microbiome variation in rice plants. The remainder of the variation is likely attributable to other metabolites, the plant immune system, and intramicrobiome regulation mechanisms. Harnessing *M* genes to engineer plant microbiomes for improved crop performance could become a new strategy in crop breeding [57]. The present study contributes to a better understanding of the complex mechanisms involved in plant genetic control of microbiome assembly.

## Supplementary Information

Supplementary Material 1. Dataset 1: Variety information for the 110 rice genotypes included in this study

Supplementary Material 2. Dataset 2: Oligonucleotide primers used in this study

Supplementary Material 3. Dataset 3: Phyllosphere microbiome analysis

Supplementary Material 4. Dataset 4: SNPs associated with metabolites in this study

Supplementary Material 5. Dataset 5: Genes associated with metabolites and microbiome

Supplementary Material 6. Dataset 6: Bacterial strains isolated from rice leaves used in this study

Supplementary Material 7. Dataset 7: Metabolite concentrations of rice leaves in this study

Supplementary Material 8

## Data Availability

Raw sequence data (16S rRNA gene fragment sequencing) generated in this study was deposited in the Genome Sequence Archive of the BIG Data Center [60], Chinese Academy of Sciences under accession PRJCA025536 [https://ngdc.cncb.ac.cn/bioproject/browse/PRJCA025536]. Taxonomic classifications of 16S rRNA gene fragments used in this study are available in the Silva v138.1 reference database [https://www.arb-silva.de/documentation/release-1381/]. Rice gene annotations used in this study are available in the Rice Annotation Project Database (RAPDB) [https://rapdb.dna.affrc.go.jp/]. All rice varieties were deposited in the State Key Laboratory of Hybrid Rice and the Institute of Plant Protection of Hunan Academy of Agricultural Sciences (Changsha, China). Other data generated in this study are provided in the Dataset files. Scripts employed in the metabolite-gene association analyses are available at Zenodo [https://zenodo.org/records/10115039]. Scripts employed in the microbiome analyses are available at Zenodo [https://zenodo.org/records/11072931].

## Abbreviations

ACN: Acetonitrile
CFA: Caffeic acid
C-RDP-II: Rice Diversity Panel II core collection
ESI: Electrospray ionization
EtOH: Ethanol
FLA: Ferulic acid
GCB: Graphitized carbon black
GWAS: Genome-Wide Association Study
HAP: Haplotype
HCA: 4-Hydroxycinnamic acid
LC-MS: Ultra-high performance liquid chromatography instrument coupled to a triple quadrupole mass spectrometer
LPA: L-phenylalanine
MLM: Mixed linear model
MRM: Multiple reaction monitoring
MS: Murashige and Skoog medium
NaOCl: Sodium hypochlorite
OD_600_: Optical density at a wavelength of 600 nm
OTU: Operational taxonomic unit
PBS: Phosphate buffer solution
PCoA: Principal coordinate analysis
PERMANOVA: Permutational Multivariate Analysis of Variance
RAPDB: Rice Annotation Project Database
RDA: Redundancy analysis
SNPs: Single-nucleotide polymorphisms
SPA: Sinapic acid
SPLT: Scopoletin

## References

[1] Liu H, Brettell LE, Qiu Z, Singh BK. Microbiome-mediated stress resistance in plants. Trends Plant Sci. 2020;25(8):733–43. 10.1016/j.tplants.2020.03.014. 32345569 10.1016/j.tplants.2020.03.014

[2] Trivedi P, Leach JE, Tringe SG, Sa T, Singh BK. Plant-microbiome interactions: from community assembly to plant health. Nat Rev Microbiol. 2020;18(11):607–21. 10.1038/s41579-020-0412-1. 32788714 10.1038/s41579-020-0412-1

[3] Huang AC, Jiang T, Liu YX, Bai YC, Reed J, Qu B, et al. A specialized metabolic network selectively modulates Arabidopsis root microbiota. Science. 2019;364(6440): eaau6389. 10.1126/science.aau6389. 31073042 10.1126/science.aau6389

[4] Zhang J, Liu YX, Zhang N, Hu B, Jin T, Xu H, et al. NRT1.1B is associated with root microbiota composition and nitrogen use in field-grown rice. Nat Biotechnol. 2019;37(6):676–84. 10.1038/s41587-019-0104-4. 31036930 10.1038/s41587-019-0104-4

[5] Shalev O, Karasov TL, Lundberg DS, Ashkenazy H, Pramoj Na Ayutthaya P, Weigel D. Commensal Pseudomonas strains facilitate protective response against pathogens in the host plant. Nat Ecol Evol. 2022;6(4):383–96. 10.1038/s41559-022-01673-7. 35210578 10.1038/s41559-022-01673-7 PMC8986537

[6] Zheng Y, Cao X, Zhou Y, Ma S, Wang Y, Li Z, et al. Purines enrich root-associated Pseudomonas and improve wild soybean growth under salt stress. Nat Commun. 2024;15(1):3520. 10.1038/s41467-024-47773-9. 38664402 10.1038/s41467-024-47773-9 PMC11045775

[7] Hawkes CV, Kjøller R, Raaijmakers JM, Riber L, Christensen S, Rasmussen S, et al. Extension of plant phenotypes by the foliar microbiome. Annu Rev Plant Biol. 2021;72:823–46. 10.1146/annurev-arplant-080620-114342. 34143648 10.1146/annurev-arplant-080620-114342

[8] Pereira LB, Thomazella DPT, Teixeira P. Plant-microbiome crosstalk and disease development. Curr Opin Plant Biol. 2023;72: 102351. 10.1016/j.pbi.2023.102351. 36848753 10.1016/j.pbi.2023.102351

[9] Wang M, Ge AH, Ma X, Wang X, Xie Q, Wang L, et al. Dynamic root microbiome sustains soybean productivity under unbalanced fertilization. Nat Commun. 2024;15(1):1668. 10.1038/s41467-024-45925-5. 38395981 10.1038/s41467-024-45925-5 PMC10891064

[10] Liu H, Li J, Singh BK. Harnessing co-evolutionary interactions between plants and Streptomyces to combat drought stress. Nat Plants. 2024;10(8):1159–71. 10.1038/s41477-024-01749-1. 39048724 10.1038/s41477-024-01749-1

[11] Wang F, Zhang H, Liu H, Wu C, Wan Y, Zhu L, et al. Combating wheat yellow mosaic virus through microbial interactions and hormone pathway modulations. Microbiome. 2024;12(1):200. 10.1186/s40168-024-01911-z. 39407339 10.1186/s40168-024-01911-z PMC11481568

[12] Harmsen N, Vesga P, Glauser G, Klötzli F, Heiman CM, Altenried A, et al. Natural plant disease suppressiveness in soils extends to insect pest control. Microbiome. 2024;12(1):127. 10.1186/s40168-024-01841-w. 39014485 10.1186/s40168-024-01841-w PMC11251354

[13] He X, Wang D, Jiang Y, Li M, Delgado-Baquerizo M, McLaughlin C, et al. Heritable microbiome variation is correlated with source environment in locally adapted maize varieties. Nat Plants. 2024;10(4):598–617. 10.1038/s41477-024-01654-7. 38514787 10.1038/s41477-024-01654-7

[14] Su P, Kang H, Peng Q, Wicaksono WA, Berg G, Liu Z, et al. Microbiome homeostasis on rice leaves is regulated by a precursor molecule of lignin biosynthesis. Nat Commun. 2024;15(1):23. 10.1038/s41467-023-44335-3. 38167850 10.1038/s41467-023-44335-3 PMC10762202

[15] Fan X, Matsumoto H, Xu H, Fang H, Pan Q, Lv T, et al. Aspergillus cvjetkovicii protects against phytopathogens through interspecies chemical signalling in the phyllosphere. Nat Microbiol. 2024;9(11):2862–76. 10.1038/s41564-024-01781-z. 39103572 10.1038/s41564-024-01781-z

[16] Chen T, Nomura K, Wang X, Sohrabi R, Xu J, Yao L, et al. A plant genetic network for preventing dysbiosis in the phyllosphere. Nature. 2020;580(7805):653–7. 10.1038/s41586-020-2185-0. 32350464 10.1038/s41586-020-2185-0 PMC7197412

[17] Bergelson J, Brachi B, Roux F, Vailleau F. Assessing the potential to harness the microbiome through plant genetics. Curr Opin Biotechnol. 2021;70:167–73. 10.1016/j.copbio.2021.05.007. 34126329 10.1016/j.copbio.2021.05.007

[18] Sohrabi R, Paasch BC, Liber JA, He SY. Phyllosphere microbiome. Annu Rev Plant Biol. 2023;74:539–68. 10.1146/annurev-arplant-102820-032704. 36854478 10.1146/annurev-arplant-102820-032704

[19] Yu P, He X, Baer M, Beirinckx S, Tian T, Moya YAT, et al. Plant flavones enrich rhizosphere Oxalobacteraceae to improve maize performance under nitrogen deprivation. Nat Plants. 2021;7(4):481–99. 10.1038/s41477-021-00897-y. 33833418 10.1038/s41477-021-00897-y

[20] Voges M, Bai Y, Schulze-Lefert P, Sattely ES. Plant-derived coumarins shape the composition of an Arabidopsis synthetic root microbiome. Proc Natl Acad Sci U S A. 2019;116(25):12558–65. 10.1073/pnas.1820691116. 31152139 10.1073/pnas.1820691116 PMC6589675

[21] Wagner MR, Lundberg DS, Del Rio TG, Tringe SG, Dangl JL, Mitchell-Olds T. Host genotype and age shape the leaf and root microbiomes of a wild perennial plant. Nat Commun. 2016;7: 12151. 10.1038/ncomms12151. 27402057 10.1038/ncomms12151 PMC4945892

[22] Deng S, Caddell DF, Xu G, Dahlen L, Washington L, Yang J, et al. Genome wide association study reveals plant loci controlling heritability of the rhizosphere microbiome. ISME J. 2021;15(11):3181–94. 10.1038/s41396-021-00993-z. 33980999 10.1038/s41396-021-00993-z PMC8528814

[23] Escudero-Martinez C, Coulter M, Alegria Terrazas R, Foito A, Kapadia R, Pietrangelo L, et al. Identifying plant genes shaping microbiota composition in the barley rhizosphere. Nat Commun. 2022;13(1):3443. 10.1038/s41467-022-31022-y. 35710760 10.1038/s41467-022-31022-y PMC9203816

[24] Oyserman BO, Flores SS, Griffioen T, Pan X, van der Wijk E, Pronk L, et al. Disentangling the genetic basis of rhizosphere microbiome assembly in tomato. Nat Commun. 2022;13(1):3228. 10.1038/s41467-022-30849-9. 35710629 10.1038/s41467-022-30849-9 PMC9203511

[25] Horton MW, Bodenhausen N, Beilsmith K, Meng D, Muegge BD, Subramanian S, et al. Genome-wide association study of Arabidopsis thaliana leaf microbial community. Nat Commun. 2014;5:5320. 10.1038/ncomms6320. 25382143 10.1038/ncomms6320 PMC4232226

[26] Chen W, Gao Y, Xie W, Gong L, Lu K, Wang W, et al. Genome-wide association analyses provide genetic and biochemical insights into natural variation in rice metabolism. Nat Genet. 2014;46(7):714–21. 10.1038/ng.3007. 24908251 10.1038/ng.3007

[27] Wang S, Alseekh S, Fernie AR, Luo J. The structure and function of major plant metabolite modifications. Mol Plant. 2019;12(7):899–919. 10.1016/j.molp.2019.06.001. 31200079 10.1016/j.molp.2019.06.001

[28] McCouch SR, Wright MH, Tung CW, Maron LG, McNally KL, Fitzgerald M, et al. Open access resources for genome-wide association mapping in rice. Nat Commun. 2016;7: 10532. 10.1038/ncomms10532. 26842267 10.1038/ncomms10532 PMC4742900

[29] Ma X, Zhang Q, Zhu Q, Liu W, Chen Y, Qiu R, et al. A robust CRISPR/Cas9 system for convenient, high-efficiency multiplex genome editing in monocot and dicot plants. Mol Plant. 2015;8(8):1274–84. 10.1016/j.molp.2015.04.007. 25917172 10.1016/j.molp.2015.04.007

[30] Dai S, Wei X, Alfonso AA, Pei L, Duque UG, Zhang Z, et al. Transgenic rice plants that overexpress transcription factors RF2a and RF2b are tolerant to rice tungro virus replication and disease. Proc Natl Acad Sci U S A. 2008;105(52):21012–6. 10.1073/pnas.0810303105. 19104064 10.1073/pnas.0810303105 PMC2634887

[31] Lin Q, Zong Y, Xue C, Wang S, Jin S, Zhu Z, et al. Prime genome editing in rice and wheat. Nat Biotechnol. 2020;38(5):582–5. 10.1038/s41587-020-0455-x. 32393904 10.1038/s41587-020-0455-x

[32] Purcell S, Neale B, Todd-Brown K, Thomas L, Ferreira MA, Bender D, et al. PLINK: a tool set for whole-genome association and population-based linkage analyses. Am J Hum Genet. 2007;81(3):559–75. 10.1086/519795. 17701901 10.1086/519795 PMC1950838

[33] Felsenstein J. PHYLIP: phylogeny inference package (Version 3.698). 2021. Accessed https://phylipweb.github.io/phylip/.

[34] Oksanen J, Simpson GL, Blanche FG, Kindt R, Legendre P, Minchin PR, et al. Vegan: community ecology package. R package version 2.6-4. 2022. Accessed https://github.com/vegandevs/vegan.

[35] Schloerke A, Cook D, Larmarange J, Briatte F, Marbach M, Thoen E, et al. GGally: extension to ‘ggplot2’. R package version 2.2.1. 2024. Accessed https://github.com/ggobi/ggally.

[36] Wickham H. ggplot2: elegant graphics for data analysis. Springer: Springer; 2016.

[37] Rognes T, Flouri T, Nichols B, Quince C, Mahe F. VSEARCH: a versatile open source tool for metagenomics. PeerJ. 2016;4: e2584. 10.7717/peerj.2584. 27781170 10.7717/peerj.2584 PMC5075697

[38] Quast C, Pruesse E, Yilmaz P, Gerken J, Schweer T, Yarza P, et al. The SILVA ribosomal RNA gene database project: improved data processing and web-based tools. Nucleic Acids Res. 2013;41((Database issue):D590-6. 10.1093/nar/gks1219. 23193283 10.1093/nar/gks1219 PMC3531112

[39] Liu YX, Qin Y, Chen T, Lu M, Qian X, Guo X, et al. A practical guide to amplicon and metagenomic analysis of microbiome data. Protein Cell. 2021;12(5):315–30. 10.1007/s13238-020-00724-8. 32394199 10.1007/s13238-020-00724-8 PMC8106563

[40] Edgar RC. Search and clustering orders of magnitude faster than BLAST. Bioinformatics. 2010;26(19):2460–1. 10.1093/bioinformatics/btq461. 20709691 10.1093/bioinformatics/btq461

[41] Edgar RC. MUSCLE: multiple sequence alignment with high accuracy and high throughput. Nucleic Acids Res. 2004;32(5):1792–7. 10.1093/nar/gkh340. 15034147 10.1093/nar/gkh340 PMC390337

[42] Price MN, Dehal PS, Arkin AP. FastTree: computing large minimum evolution trees with profiles instead of a distance matrix. Mol Biol Evol. 2009;26(7):1641–50. 10.1093/molbev/msp077. 19377059 10.1093/molbev/msp077 PMC2693737

[43] Sakai H, Lee SS, Tanaka T, Numa H, Kim J, Kawahara Y, et al. Rice Annotation Project Database (RAP-DB): an integrative and interactive database for rice genomics. Plant Cell Physiol. 2013;54(2): e6. 10.1093/pcp/pcs183. 23299411 10.1093/pcp/pcs183 PMC3583025

[44] Zhang X, Liu CJ. Multifaceted regulations of gateway enzyme phenylalanine ammonia-lyase in the biosynthesis of phenylpropanoids. Mol Plant. 2015;8(1):17–27. 10.1016/j.molp.2014.11.001. 25578269 10.1016/j.molp.2014.11.001

[45] Knosp S, Kriegshauser L, Tatsumi K, Malherbe L, Erhardt M, Wiedemann G, et al. An ancient role for CYP73 monooxygenases in phenylpropanoid biosynthesis and embryophyte development. EMBO J. 2024;43(18):4092–109. 10.1038/s44318-024-00181-7. 39090438 10.1038/s44318-024-00181-7 PMC11405693

[46] He J, Liu Y, Yuan D, Duan M, Liu Y, Shen Z, et al. An R2R3 MYB transcription factor confers brown planthopper resistance by regulating the phenylalanine ammonia-lyase pathway in rice. Proc Natl Acad Sci U S A. 2020;117(1):271–7. 10.1073/pnas.1902771116. 31848246 10.1073/pnas.1902771116 PMC6955232

[47] Compant S, Cassan F, Kostić T, Johnson L, Brader G, Trognitz F, et al. Harnessing the plant microbiome for sustainable crop production. Nat Rev Microbiol. 2025;23(1):9–23. 10.1038/s41579-024-01079-1. 39147829 10.1038/s41579-024-01079-1

[48] Zhong Y, Xun W, Wang X, Tian S, Zhang Y, Li D, et al. Root-secreted bitter triterpene modulates the rhizosphere microbiota to improve plant fitness. Nat Plants. 2022;8(8):887–96. 10.1038/s41477-022-01201-2. 35915145 10.1038/s41477-022-01201-2

[49] Stringlis IA, Yu K, Feussner K, de Jonge R, Van Bentum S, Van Verk MC, et al. MYB72-dependent coumarin exudation shapes root microbiome assembly to promote plant health. Proc Natl Acad Sci U S A. 2018;115(22):E5213–22. 10.1073/pnas.1722335115. 29686086 10.1073/pnas.1722335115 PMC5984513

[50] Matsumoto H, Fan X, Wang Y, Kusstatscher P, Duan J, Wu S, et al. Bacterial seed endophyte shapes disease resistance in rice. Nat Plants. 2021;7(1):60–72. 10.1038/s41477-020-00826-5. 33398157 10.1038/s41477-020-00826-5

[51] Chandan RK, Kumar R, Kabyashree K, Yadav SK, Roy M, Swain DM, et al. A prophage tail-like protein facilitates the endophytic growth of Burkholderia gladioli and mounting immunity in tomato. New Phytol. 2023;240(3):1202–18. 10.1111/nph.19184. 37559429 10.1111/nph.19184

[52] Liu D, He J, Li Q, Zhang X, Wang Y, Sun Q, et al. A WRKY transcription factor confers broad-spectrum resistance to biotic stresses and yield stability in rice. Proc Natl Acad Sci U S A. 2025;122(10): e2411164122. 10.1073/pnas.2411164122. 40042898 10.1073/pnas.2411164122 PMC11912400

[53] Zeng Y, Lu X, Wang M, Chen R, Li Q, Zhu J, et al. Endophyte Acrocalymma vagum establishes the holobiont with rice to attract beneficial microorganisms and promote disease resistance. J Adv Res. 2025. 10.1016/j.jare.2025.03.008.40054578 10.1016/j.jare.2025.03.008

[54] Batool M, Carvalhais LC, Fu B, Schenk PM. Customized plant microbiome engineering for food security. Trends Plant Sci. 2024;29(4):482–94. 10.1016/j.tplants.2023.10.012. 37977879 10.1016/j.tplants.2023.10.012

[55] Zhan C, Matsumoto H, Liu Y, Wang M. Pathways to engineering the phyllosphere microbiome for sustainable crop production. Nat Food. 2022;3(12):997–1004. 10.1038/s43016-022-00636-2. 37118297 10.1038/s43016-022-00636-2

[56] Zhan C, Wang M. Disease resistance through M genes. Nat Plants. 2024;10(3):352–3. 10.1038/s41477-024-01644-9. 38409293 10.1038/s41477-024-01644-9

[57] Cernava T. Coming of age for microbiome gene breeding in plants. Nat Commun. 2024;15(1):6623. 10.1038/s41467-024-50700-7. 39103326 10.1038/s41467-024-50700-7 PMC11300713

[58] Howe A, Stopnisek N, Dooley SK, Yang F, Grady KL, Shade A. Seasonal activities of the phyllosphere microbiome of perennial crops. Nat Commun. 2023;14(1):1039. 10.1038/s41467-023-36515-y. 36823152 10.1038/s41467-023-36515-y PMC9950430

[59] Zhernakova DV, Wang D, Liu L, Andreu-Sánchez S, Zhang Y, Ruiz-Moreno AJ, et al. Host genetic regulation of human gut microbial structural variation. Nature. 2024;625(7996):813–21. 10.1038/s41586-023-06893-w. 38172637 10.1038/s41586-023-06893-w PMC10808065

[60] Database resources of the BIG Data Center in 2018. Nucleic Acids Res. 2018;46(D1):D14-d20. 10.1093/nar/gkx897. 10.1093/nar/gkx897 PMC5753194 29036542

