# Supplementary material for: Host metabolites explain microbiome variation between different rice genotypes
Source: Microbiome. 2025 Aug 9;13:185. doi: 10.1186/s40168-025-02181-z (PMC12335145; doi:10.1186/s40168-025-02181-z)
Supplement: Supplementary file 9 — Supplementary Material 8 [file 40168_2025_2181_MOESM8_ESM.docx]

**Supporting Information for:**

Host metabolites explain microbiome variation between different rice genotypes

Pin Su^1^**^#^**, Houxiang Kang^2^**^#^**, Qianze Peng^3,4^**^#^**, Weiye Peng^1^**^#^**, Shu'e Sun^1^, Xiaohua Du^1^, Chi Zhang^5^, Ziling Lei^5^, Lianyang Bai^1^, Qianjun Tang^6^，Yong Liu^1,5*^, Tomislav Cernava^7*^, and Deyong Zhang^1,3,4,5*^

* To whom correspondence may be addressed.

**Email:** zhangdeyong@hhrrc.ac.cn, t.cernava@soton.ac.uk, liuyong@hunaas.cn


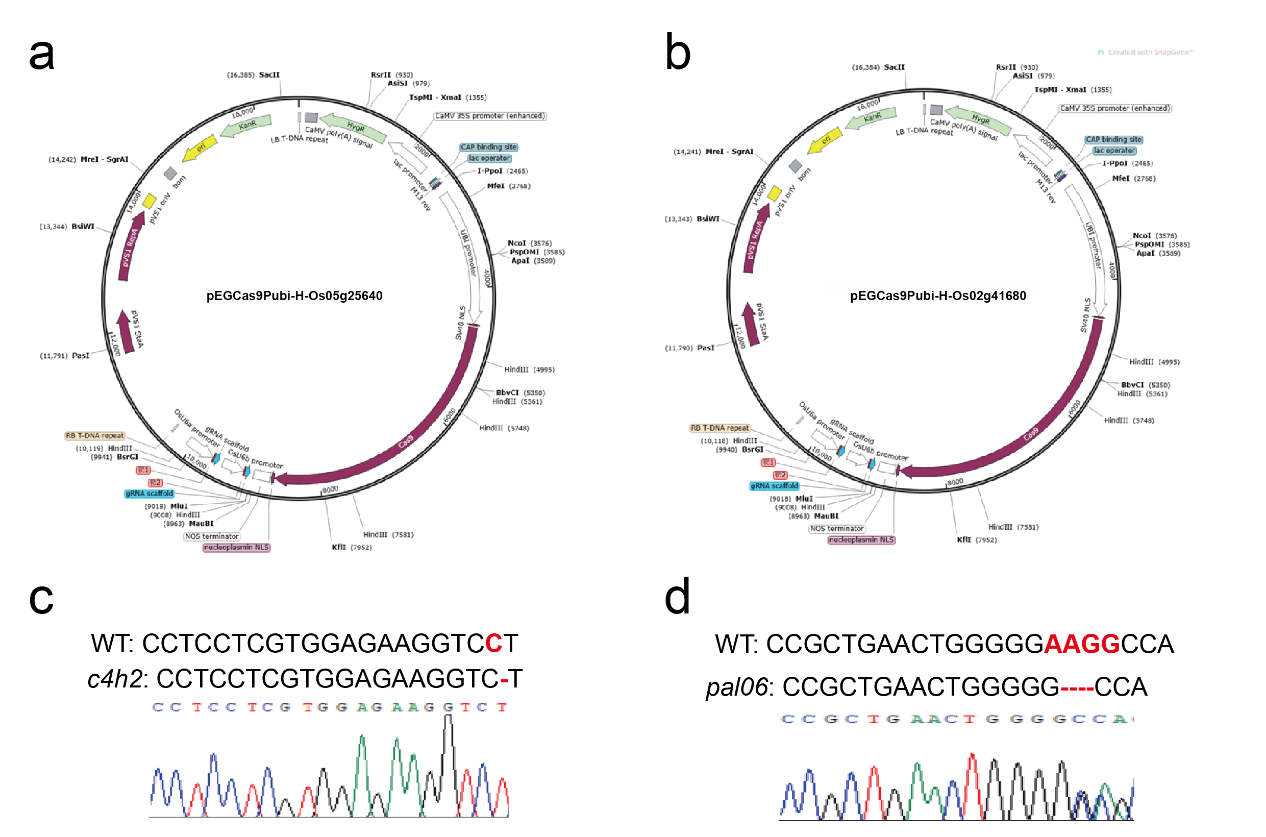


Supplementary Fig. 1. Generation of *OsC4H2* (Os05g25640) and *OsPAL06* (Os02g41680) KO plants.

**a**, The vector pEGCas9Pubi-H-Os05g25640 was used to generate CRISPR–Cas9 edited rice plants. **b**, The vector pEGCas9Pubi-H-Os02g41680 was used to generate CRISPR–Cas9 edited rice plants. **c**, Sequences of *OsC4H2*-KO plants at CRISPR–Cas9 target site. **d**, Sequences of *OsPAL06*-KO plants at CRISPR–Cas9 target site.

­
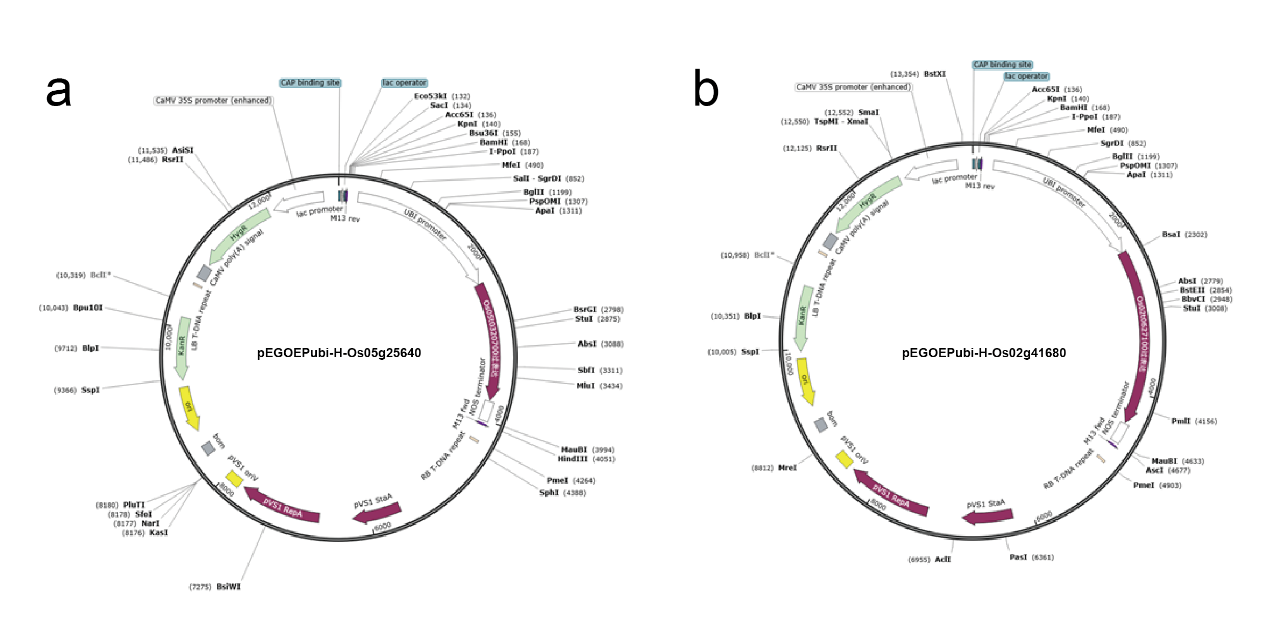


Supplementary Fig. 2. Generation of *OsC4H2* (Os05g25640) and *OsPAL06* (Os02g41680) OE plants.

**a**, The vector pEGOEPubi-H-Os05g25640 was used to generate *OsC4H2*-overexpression rice plants. **b**, The vector pEGOEPubi-H-Os02g41680 was used to generate *OsPAL06*-overexpression rice plants.


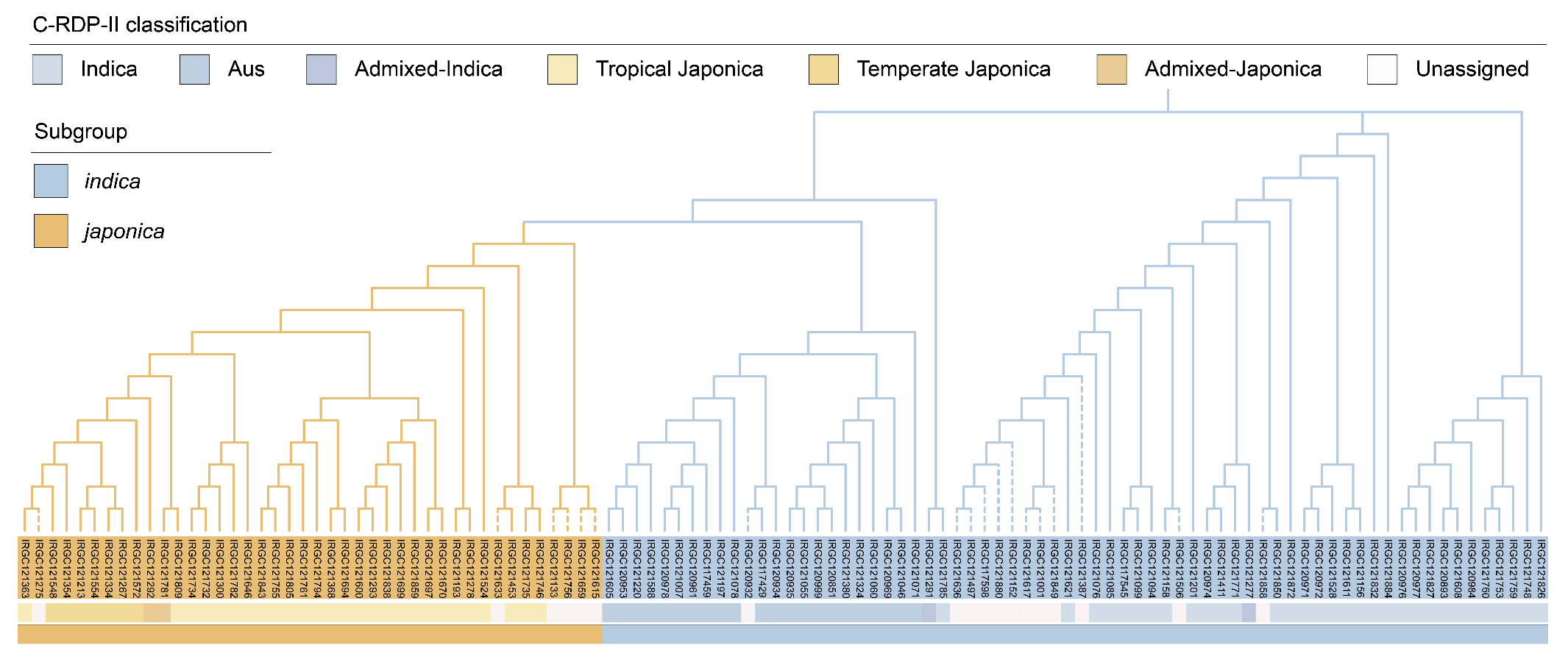


Supplementary Fig. 3. Phylogenetic analysis of 110 rice varieties.

Dashed lines and solid lines indicate unclassified and classified varieties within C-RDP-II, respectively. The middle color strip represents the subpopulation-level classification of the C-RDP-II collection of 110 rice varieties. The bottom color strip represents *indica* and *japonica* subgroups based on classified rice varieties and phylogenetic distance.


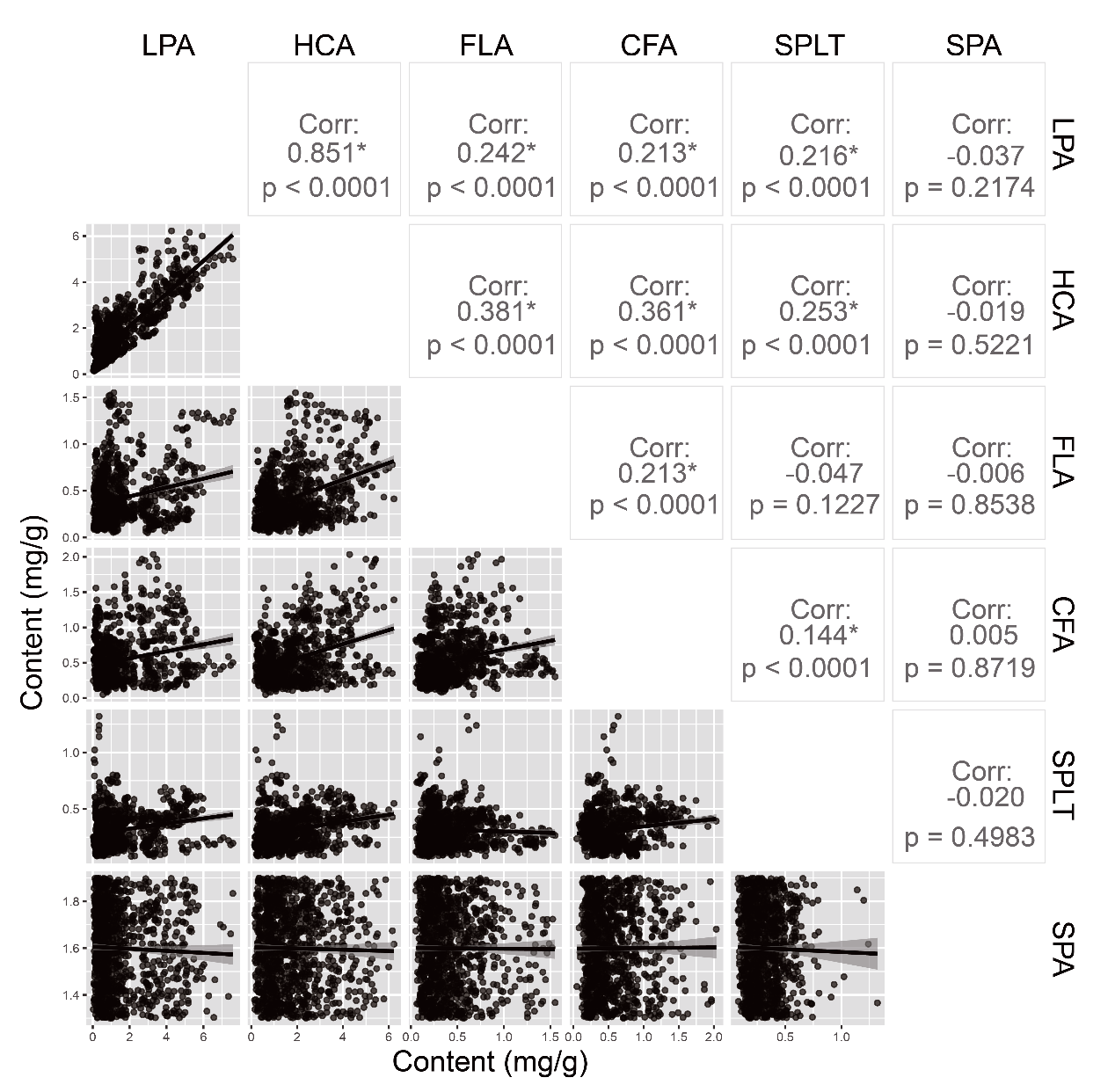


Supplementary Fig. 4. Correlation analyses between different leaf metabolites.

Two-sided Pearson coefficient R and p-values were calculated using GGally. The grey area shows the 95% confidence interval of the regression line (black line). HCA, LPA, FLA, CFA, SPA and SPLT indicate 4-hydroxycinnamic acid, L-phenylalanine, ferulic acid, caffeic acid, sinapic acid, and scopoletin, respectively. The number of replicated samples for each metabolite are 110 rice varieties with ten replications for each genotype.


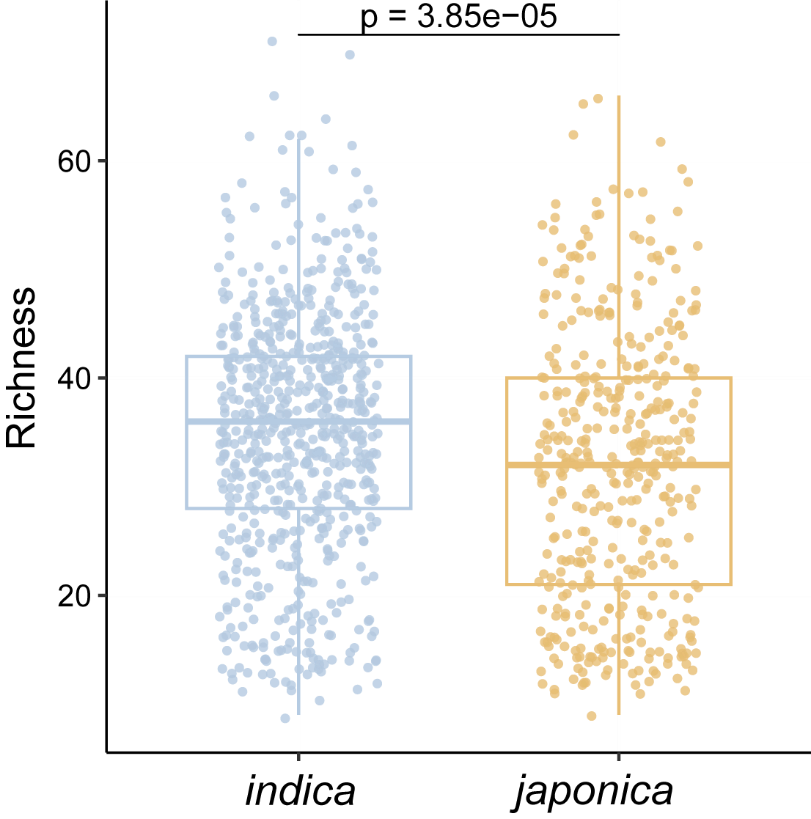


Supplementary Fig. 5. Richness for phyllosphere bacterial communities of *indica* and *japonica* varieties.

The horizontal bars within boxes represent medians. The tops and bottoms of boxes represent the 75th and 25th percentiles, respectively. The upper and lower whiskers extend to data no more than 1.5× the interquartile range from the upper edge and lower edge of the box, respectively. The p-value was calculated with unpaired two-tailed Student’s t-test. In this figure, *indica* (n=68) and *japonica* (n=42) rice samples were included with ten replications for each genotype.


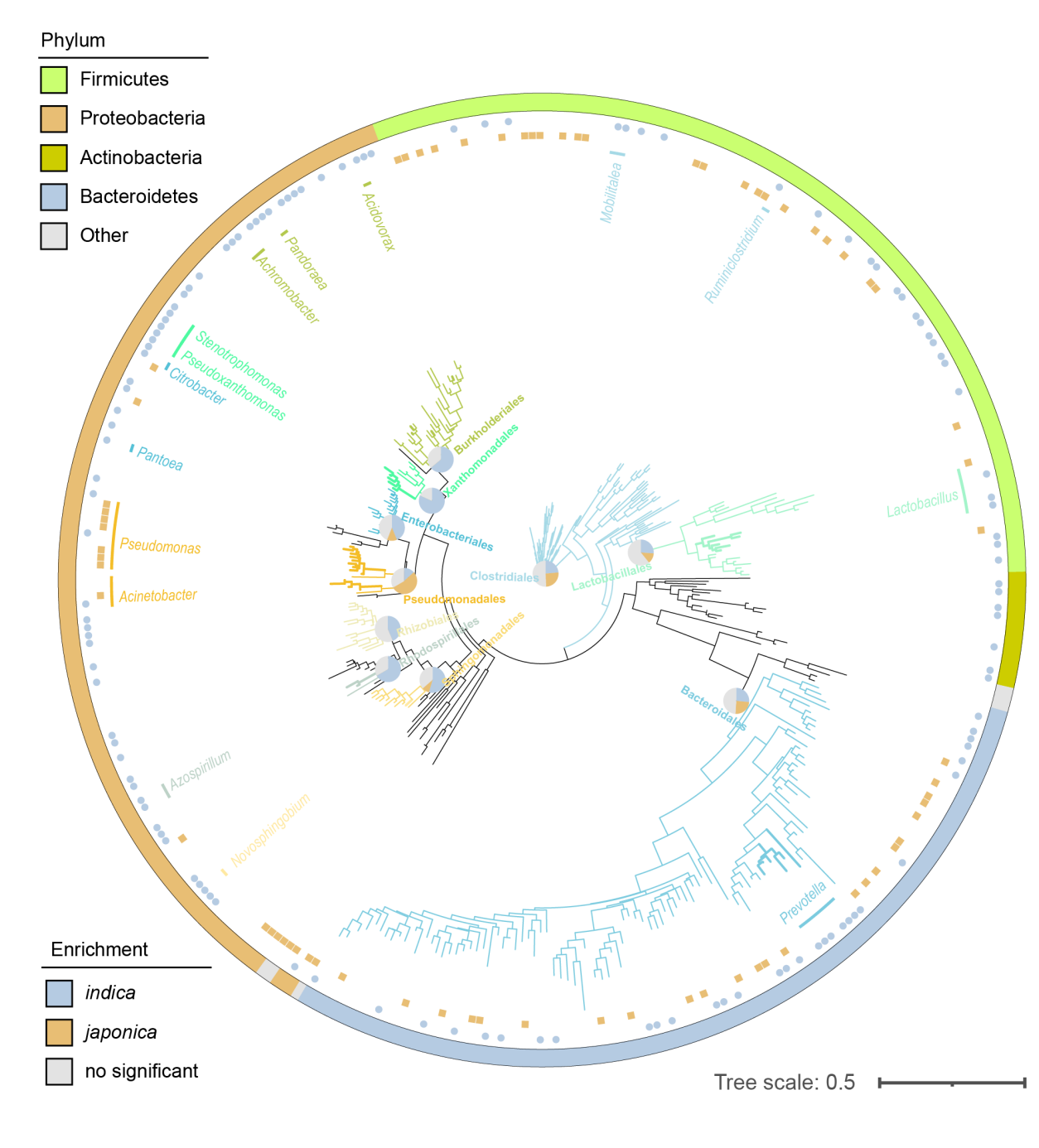


Supplementary Fig. 6. Phylogenetic tree of the rice phyllosphere microbiome.

The inner part represents the phylogeny of all bacterial OTUs (Operational Taxonomic Units) with a relative abundance > 0.02% in *indica* and *japonica* phyllosphere microbiomes. Different colors of branches and text indicate order-level classification of OTUs. The pie chart indicates OTU proportions with significant enrichment in *indica* or *japonica*. Squares and circles represent OTUs with significant abundance differences in *japonica* and *indica* rice varieties, respectively. The p-value was calculated with unpaired two-tailed Student’s t-test (p-values are listed in Dataset 3). *Indica* (n=68) and *japonica* (n=42) plants with ten replications for each genotype were analyzed. The outer ring represents phylum-level classification of OTUs.


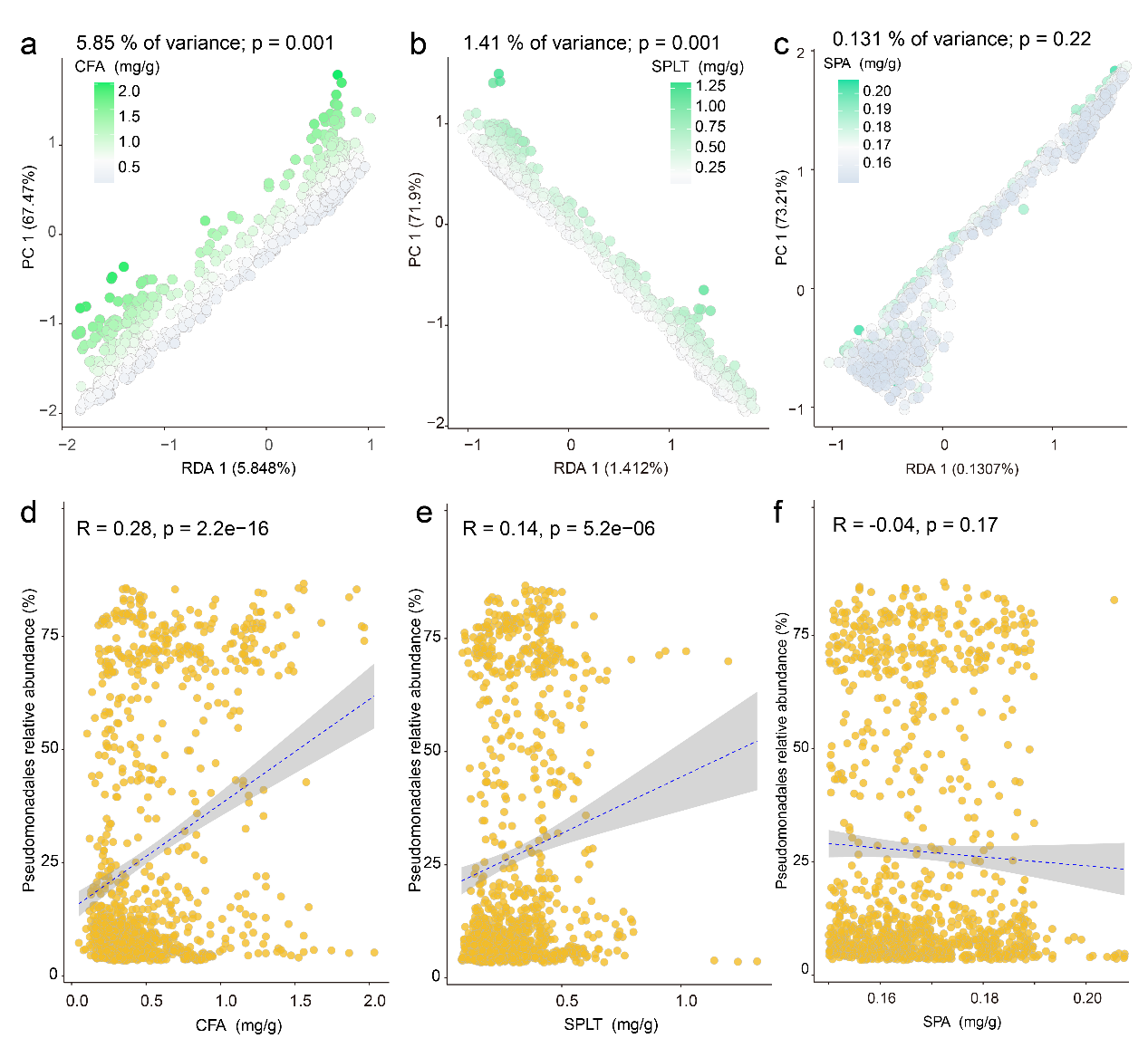


Supplementary Fig. 7. Association analysis between metabolites and phyllosphere microbiome.

**a-c**, Phyllosphere microbiome variance explained by CFA (caffeic acid, **a**, 5.85% of total variance, p = 0.001), SPLT (scopoletin, **b**, 1.41% of total variance, p = 0.001) and SPA (sinapic acid, **c**, explained 0.131% of total variance, p = 0.22), respectively. The p-value was calculated through one-way PERMANOVA; different colors indicate concentration levels of the metabolites. **d**-**f,** Correlation analysis between relative abundance of Pseudomonadales and CFA (**d**), SPLT (**e**) and SPA (**f**) concentrations in rice leaves, respectively. The two-sided Pearson coefficient R and p-values were calculated using ggplot2; the grey area shows the 95% confidence interval of the regression line (blue dashed line). A total of 110 rice varieties with ten replications for each genotype were analyzed.


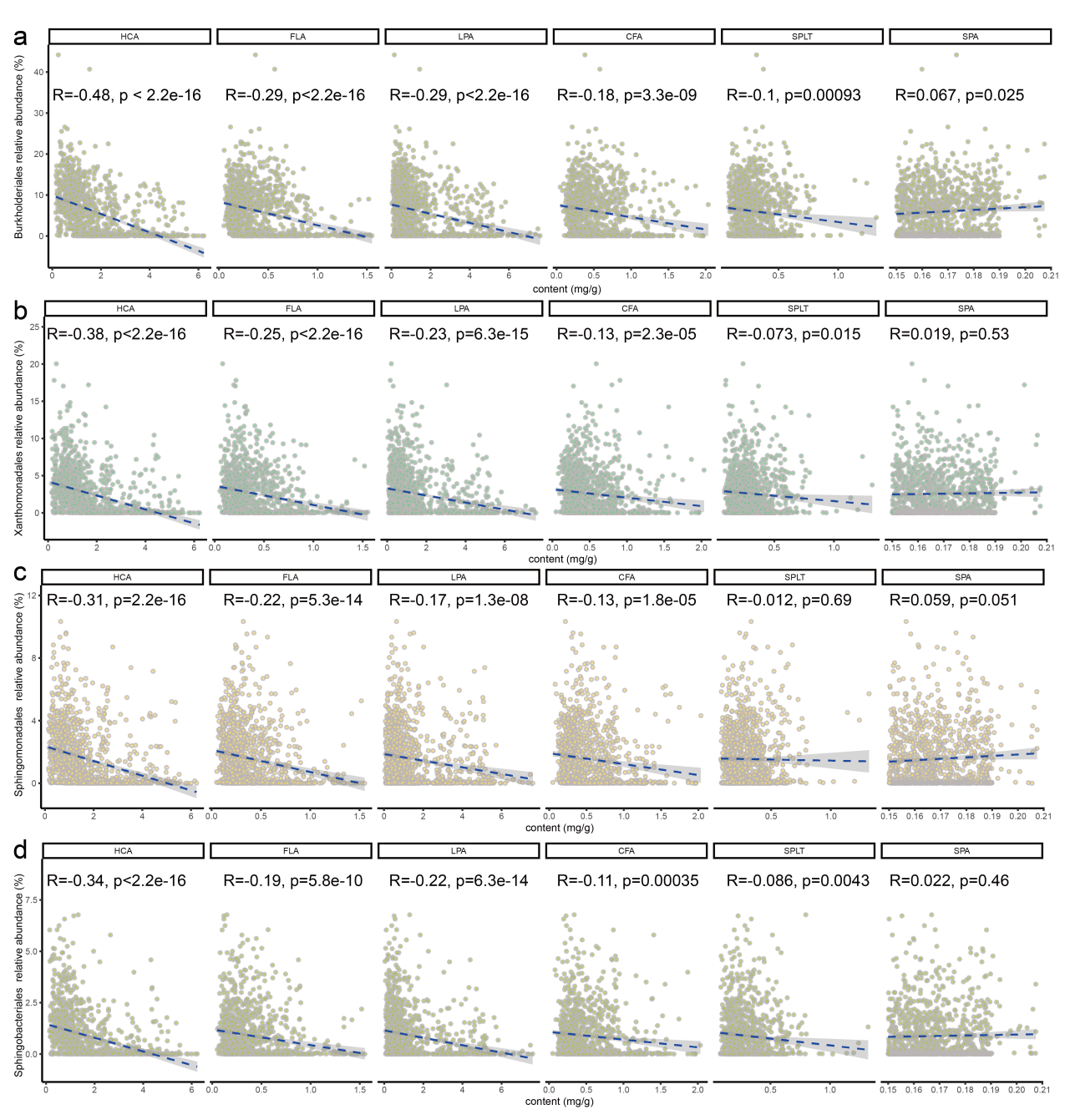


Supplementary Fig. 8. Correlation analysis between relative abundances of bacterial orders and metabolite concentrations.

**a**-**d**, Correlation analysis between relative abundance of Burkholderiales (**a**), Xanthomonadales (**b**), Sphingomonadales (**c**), Sphingobacteriales (**d**) and HCA (4-hydroxycinnamic acid), FLA (ferulic acid), LPA (L-phenylalanine), CFA (caffeic acid), SPLT (scopoletin), and SPA (sinapic acid) concentrations in rice leaves, respectively. The two-sided Pearson coefficient R and p-values were calculated using ggplot2; the grey area shows the 95% confidence interval of the regression line (blue dashed line). The analysis was conducted with 110 rice varieties and ten replications for each genotype.


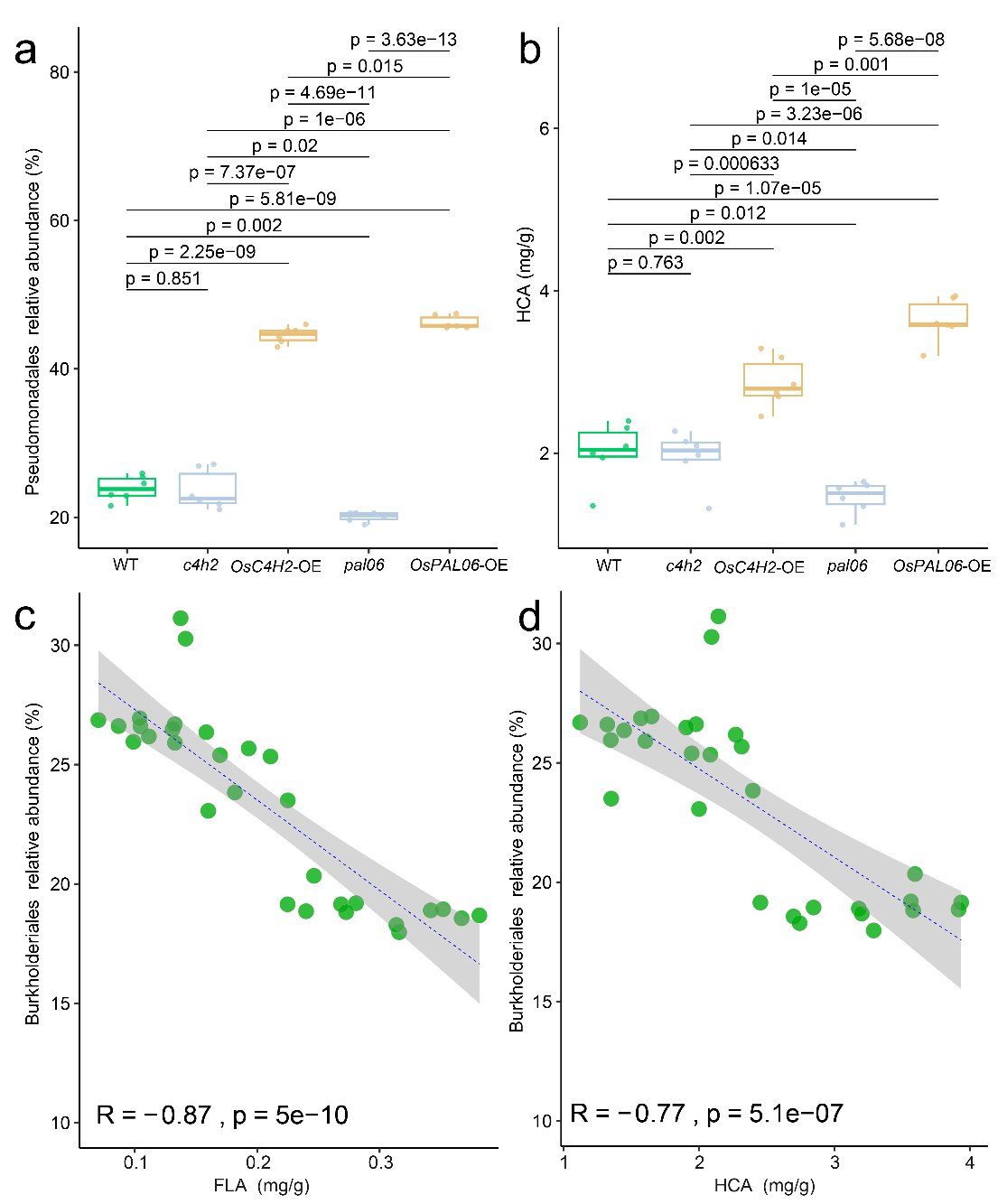


Supplementary Fig. 9. *OsC4H2* and *OsPAL06* regulate phyllosphere microbiome and metabolites.

**a-b,** Comparison of relative abundances of Pseudomonadales (**a**) and concentrations of HCA (4-hydroxycinnamic acid, **b**) between WT, *c4h2*, *OsC4H2*-OE, *pal06* and *OsPAL06*-OE plants. The numbers of replicated samples are as follows: WT (n=6), *c4h2*, (n=6), *OsC4H2*-OE (n=6), *pal06* (n=6) and *OsPAL06*-OE (n=6). The horizontal bars within boxes represent medians. The tops and bottoms of boxes represent the 75th and 25th percentiles, respectively. The upper and lower whiskers extend to data no more than 1.5× the interquartile range from the upper edge and lower edge of the box, respectively. The p-value was calculated with unpaired two-tailed Student’s t-test. **c**-**d,** Correlation analysis between relative abundance of Burkholderiales and FLA (ferulic acid, **c**) and HCA (**d**) concentrations in rice leaves, respectively. The two-sided Pearson coefficient R and p-values were calculated using ggplot2; the grey area shows the 95% confidence interval of the regression line (blue dashed line). WT, *c4h2*, *OsC4H2*-OE, *pal06* and *OsPAL06*-OE plants with six replications were analyzed.


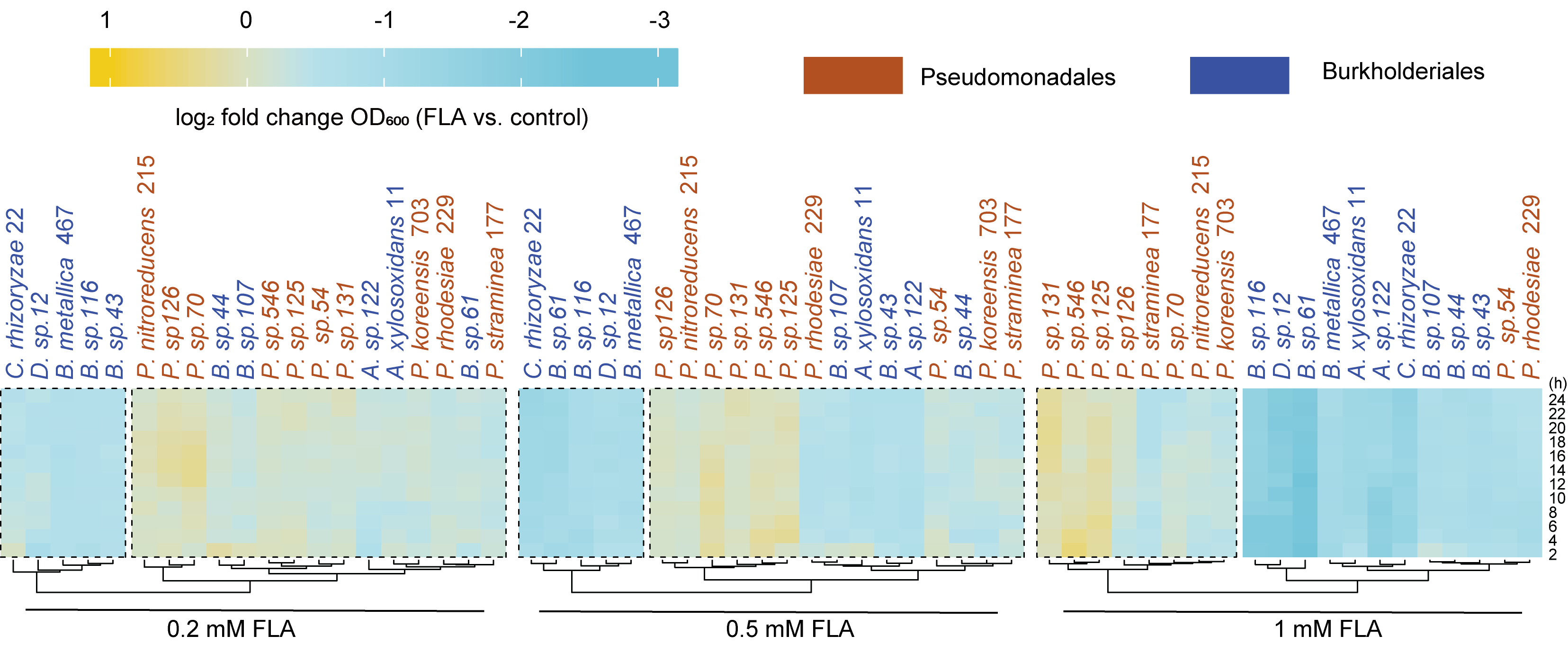


Supplementary Fig. 10. Effect of FLA on growth of bacterial isolates from the orders Burkholderiales and Pseudomonadales.

The heatmap shows log_2_ fold changes (OD_600_) of individual isolates exposed to 0.2 mM, 0.5 mM and 1 mM FLA versus control over 24 h, respectively. Different colors represent isolates assigned to Burkholderiales and Pseudomonadales, respectively. Clusters are shown on the bottom using Euclidean distance.

Supplementary Table 1. MS settings for the detection of leaf metabolites in rice plants.

| **Components** | **Ionization mode** | **Precursor ion (m/z)** | **Product ion (CE), m/z** | | **CV(V)** |
| --- | --- | --- | --- | --- | --- |
|  |  |  | **Quantitation** | **Confirmation** |  |
| LPA | ESI+ | 166.1 | 120.1(15) | 103.1(25) | 25 |
| SPLT | ESI+ | 193.0 | 133.0(20) | 122.0(30) | 40 |
| FLA | ESI+ | 195.1 | 177.1(10) | 145.1(15) | 25 |
| HCA | ESI- | 163.1 | 119.1(-20) | 93.1(-300) | 30 |
| CFA | ESI- | 179.1 | 135.1(-15) | 107.1(-20) | 35 |
| SPA | ESI- | 223.1 | 164.1(-15) | 149.1(-20) | 30 |

**Note:** CE indicates collision energy (eV). HCA, LPA, FLA, CFA, SPA and SPLT indicate 4-hydroxycinnamic acid, L-phenylalanine, ferulic acid, caffeic acid, sinapic acid, and scopoletin, respectively.

Supplementary Table 2. Standard curves and recovery rates for the targeted leaf metabolites.

| **Components** | **linear** **equations** | **R^2^** | **Average recovery rate (%)** |
| --- | --- | --- | --- |
| LPA | y = 32.237x - 159.55 | R² = 0.9993 | 78.2 |
| SPLT | y = 49.471x - 187.1 | R² = 0.9989 | 71.7 |
| FLA | y = 36.79x - 34.881 | R² = 0.9998 | 73.5 |
| HCA | y = 52.679x + 607.26 | R² = 0.9986 | 80.7 |
| CFA | y = 57.594x - 1234.9 | R² = 0.9907 | 72.1 |
| SPA | y = 18.16x - 167.57 | R² = 0.9957 | 70.4 |

**Note:** The linear equations were calculated with ggplot2. The number of replicated samples for construction of the standard curves and recovery tests is ten replications for each metabolite. HCA, LPA, FLA, CFA, SPA and SPLT indicate 4-hydroxycinnamic acid, L-phenylalanine, ferulic acid, caffeic acid, sinapic acid, and scopoletin, respectively.

Supplementary Table 3. Assessment of differences in metabolite concentrations between *indica* and *japonica* varieties.

| **Metabolite** | ***indica*** | ***japonica*** | **p-value** | **Difference (%)** |
| --- | --- | --- | --- | --- |
| HCA | 1.129±0.77 | 2.736±1.23 | 8.46E-91 | 242.34 |
| SPLT | 0.277±0.16 | 0.375±0.13 | 5.25E-28 | 135.38 |
| CFA | 0.487±0.27 | 0.623±0.43 | 7.53E-09 | 127.93 |
| LPA | 0.960±0.98 | 2.417±1.73 | 4.90E-47 | 251.77 |
| FLA | 0.367±0.25 | 0.498±0.34 | 1.95E-11 | 135.69 |
| SPA | 0.169±0.01 | 0.167±0.01 | 0.00386 | 98.82 |

**Note:** The p-values were calculated with unpaired two-tailed Student’s t-test. Values are means ± SD of metabolite concentrations (mg/g). The numbers of replicated samples are as follows: *indica* (n=68) and *japonica* (n=42) with ten replications for each genotype. Difference indicates the relative concentration in *japonica* accessions as compared with *indica* accessions. HCA, LPA, FLA, CFA, SPA and SPLT indicate 4-hydroxycinnamic acid, L-phenylalanine, ferulic acid, caffeic acid, sinapic acid, and scopoletin, respectively.
